# Experiences of Food Insecurity During Pregnancy in High‐Income Countries: A Meta‐Synthesis of Qualitative Studies

**DOI:** 10.1111/jhn.70244

**Published:** 2026-04-13

**Authors:** Steph Scott, Giang Nguyen, Zoë Bell, Lucy Clark, Paige van der Pligt, Fiona H. McKay, Julia Zinga, Nicola Heslehurst

**Affiliations:** ^1^ Baddiley Clark Building, Population Health Sciences Institute, Faculty of Medical Sciences, Newcastle University Newcastle upon Tyne UK; ^2^ Department of Nutritional Sciences King's College London, Franklin‐Wilkins Building London UK; ^3^ Population Health Sciences Institute, Faculty of Medical Sciences, Newcastle University Newcastle upon Tyne UK; ^4^ Department of Allied Health School of Health Sciences, Swinburne University of Technology Melbourne Victoria Australia; ^5^ School of Health and Social Development and Institute for Health Transformation, Faculty of Health, Deakin University Burwood Victoria Australia; ^6^ Royal Women's Hospital Parkville Victoria Australia

**Keywords:** diet, food insecurity, meta‐synthesis, nutrition, pregnancy, qualitative research, systematic review

## Abstract

**Introduction:**

Food insecurity, when individuals do not have sufficient access to food, has rapidly increased in high‐income countries (HICs) since the 2008 global financial crisis. Women are particularly at risk of experiencing food insecurity, and during pregnancy, this can have detrimental physical and emotional health implications.

**Objective:**

To synthesise qualitative research exploring pregnant women's experiences of food insecurity in HICs (PROSPERO 2023 CRD42023404774).

**Methods:**

Systematic review of qualitative literature reporting data on women's experiences of food insecurity. Six databases (MEDLINE, Embase, Web of Science, CINAHL, ASSIA, Scopus) and grey literature sources were searched, followed by forwards and backwards citation chaining for all included studies. Screening of titles, abstracts and full‐texts, data extractions and quality appraisals (using the Critical Appraisal Skills Programme (CASP) Qualitative Studies Checklist) were completed in duplicate. Certainty in the evidence was evaluated using GRADE‐CERQual.

**Participants/Setting:**

Food‐insecure pregnant and postnatal women, in HICs, since the global financial crisis of 2008.

**Main Outcome Measures:**

Experiences of food insecurity during pregnancy.

**Analyses:**

Thematic synthesis using NVivo14 to code data. Hand‐drawn thematic maps were used to group codes into sub‐themes and overarching themes. Coding and hand‐drawn thematic maps were combined to create a final visual summary of analytical themes.

**Results:**

Searches resulted in 32,685 studies, and 32 were included (*n* = 20 North America, *n* = 10 Europe, *n* = 2 Australia). Findings identified three overarching themes: (1) barriers in access to food, (2) impact on physical and mental health, and (3) established individual, informal and statutory coping strategies. Women frequently discussed barriers to accessing fresh fruit and vegetables, resulting in poorer quality diets. Whilst qualitative data extracted precluded any direct pregnancy versus pre‐pregnancy comparison, pregnancy appeared to exacerbate the experiences of food insecurity for women. The future arrival of a newborn created additional financial concerns along with worries over nutritional needs. Reliance on others was a recurrent strategy for pregnant women to mitigate the impact of food insecurity. The GRADE CERQual assessment showed moderate to high confidence in all findings.

**Conclusions:**

The findings of this qualitative review—the first to focus on experiences of food insecurity during pregnancy across HICs—show that women are experiencing substantial impacts from food insecurity during this critical life course stage. Review findings emphasise the need for co‐ordinated screening and interventions that aim to support women to mitigate the impacts of food insecurity and its underlying causes to improve postpartum health and wellbeing.

## Summary

- This is the first qualitative systematic review to summarise the qualitative experiences of pregnant women who are food insecure within High Income Countries (HICs) since the global financial crisis of 2008.
- Data synthesised from 32 studies spanning North America, Europe and Australia highlighted three interlinking themes, encompassing the impact of economic, physical and environmental barriers that prohibited optimal antenatal nutrition, the resultant physical and mental health concerns that women held for themselves and their baby, and their necessity to establish coping mechanisms. We identified no empirical qualitative literature capturing the experiences of women in non‐Western HIC contexts.
- Findings from our meta‐synthesis reinforce the need for co‐ordinated screening and interventions to support women to mitigate the impacts of food insecurity and its underlying causes to improve postpartum health and wellbeing.

## 1 Introduction

Food insecurity is the “limited or uncertain availability of nutritionally adequate and safe foods, acquired in socially acceptable ways” [1]. Food security is conditional upon a range of factors, most recently conceptualised as a six‐dimensional framework: *availability*, *access, utilisation, stability, sustainability* and *agency* [2]. Thus, adequate food availability does not guarantee access to sufficient, safe and nutritious food, a phenomenon seen particularly in high‐income countries (HICs) where food is often available but difficult to access in circumstances of financial hardship or material deprivation [3, 4, 5]. Thus, whilst rates of food insecurity are historically higher in LMICs [6], the prevalence of food insecurity in HICs is increasing, with reported rates ranging between 8% and 20% [7]. Most recently, the cost‐of‐living crisis, COVID‐19 pandemic, the 2008 global financial crisis, current global conflicts, and increased fuel costs have all placed additional pressures upon household food budgets, heightening the risks and experiences of food insecurity in HICs [8, 9, 10]. As a consequence, low‐income households in HICs, who spend a large proportion of their income on food, have experienced a decline in their nutritional status having shifted their eating behaviours to include more energy‐rich and nutritionally poor foods that are more accessible and often more affordable [11].

During pregnancy, diet and nutrition have great importance and food insecurity during pregnancy has been linked to a range of negative health outcomes, such as obesity, gestational diabetes, increased pregnancy‐related mortalities (both maternal deaths and neonatal death/stillbirth), and poor mental health [12, 13, 14, 15]. Meanwhile, women remain unfavourably affected by long‐established gender inequalities and socio‐economic factors that make them more susceptible to food insecurity compared to men [16, 17]. These factors can include poor employment and education, domestic violence, lone parenting, and working part‐time or being unemployed due to caring responsibilities [17, 18].

It is important to understand pregnant women's experiences of food insecurity in addition to recognising what is already known from quantitative data about health impacts and health outcomes associated with food insecurity. Understanding pregnant women's experiences will shed light on the strategies they use to manage, adjust, and cope with food insecurity, and provide nuance to understanding their complex and different situations. However, there is a significant gap in reviews focusing on pregnant women's experiences of food insecurity in HICs. Previous systematic reviews exploring food insecurity experiences have focused on migrants and refugees, homeless individuals, students, children, or non‐pregnant women [19, 20, 21, 22, 23]. Meanwhile, one further review investigating pregnant women's experiences comprised studies conducted in LMICs [24]. Therefore, this systematic review aimed to explore the experiences of food‐insecure pregnant women in HICs, using qualitative data, in the period after the 2008 global financial crisis.

## 2 Methods

This systematic review of qualitative studies was conducted following the Enhancing Transparency in Reporting the Synthesis of Qualitative research (ENTREQ) reporting guidelines [25]. The protocol for this review was prospectively registered with PROSPERO (CRD42023404774) [26].

### 2.1 Data Sources and Searches

A rigorous search strategy was developed in partnership with a medical sciences librarian at Newcastle University (Supporting Information S1: Table S1). Databases searched were MEDLINE, Embase, Scopus, Cumulative Index to Nursing and Allied Health Literature (CINAHL), Web of Science, and Applied Social Science Index and Abstracts (ASSIA) from 2008 to 16 January 2026 (Supporting Information S1: Table S1). The websites searched for grey literature were The Food Foundation [27], The Trussell Trust [28], The Kings Fund [29] and the World Health Organisation [30] (Supporting Information S1: Table S2). All included studies were subjected to reference lists and citation searches to supplement the database searches. Citation chaining and contacting authors (when further information was required for analysis) were completed in February 2026. Citation chaining searches (forward and backwards facing reference and citation searches) were conducted for all included studies using R‐Shinny app [31] in February 2026.

### 2.2 Study Selection and Screening

Titles and abstracts from identified studies were imported into EndNote 20 [32] for deduplication and Rayyan [33] for duplicate blind screening involving all co‐authors. Any studies that potentially met the inclusion criteria were taken forward to full‐text duplicate blind screening against the inclusion criteria. All full‐text screening was conducted independently, in duplicate, with any conflicts resolved between reviewers. Any screening disagreements were resolved by discussion. A PRISMA flow chart was used to record each step involved in the searching, screening and selection of eligible studies [34].

Inclusion and exclusion criteria were developed using the SPIDER framework for searching qualitative research [35]: Sample (S), Phenomenon of Interest (PI), Design (D), Evaluation (E), and Research type (R). Qualitative primary research studies published in peer‐reviewed journals or grey literature sources that reported data collected from 2008 onwards in HICs (defined by World Bank criteria [36]) (D and R) on the experiences (E) of food insecurity (PI) during pregnancy among pregnant and post‐natal women (S) were included; there were no restrictions on qualitative study design. Food insecurity was defined as being either explicit (e.g., women had been screened for food insecurity) or implied (e.g., low‐income pregnant women describing their experiences of any of the pillars of food insecurity), whereas studies that reported barriers to healthy eating not associated with low‐income status or living in poverty were excluded. There were no restrictions on study reporting language. To mitigate language bias, Google translate was used to assess whether titles and abstracts of non‐English language studies screened met the inclusion criteria. Quantitative studies, conference abstracts, grey literature not reporting empirical data from primary studies, editorials, or studies reporting data on general population experiences of food insecurity were excluded.

### 2.3 Data Extraction and Quality Appraisal

Data extraction and quality appraisal were conducted in duplicate for all included studies. A data extraction template was developed in Microsoft Excel, tailored to the review question and comprising the following fields: study characteristics (country of study, setting for recruitment, time period of study, whether food insecurity was explicit or implied, aims and objectives); methodology characteristics (study design, inclusion and exclusion criteria, methods of data collection and analysis, sample size); population characteristics (whether the population included just pregnant women or pregnant and postnatal women, age, ethnic group, marital status, single parent household, employment, migration, education, income or socio‐economic position, parity); and the author interpretation of key themes and recommendations relating to women's experiences of food insecurity during pregnancy. The Critical Appraisal Skills Programme (CASP) Qualitative Studies Checklist was used to complete quality assessment of all included studies (Supporting Information S1: Table S3) [37]. This appraisal tool is made up of 10 questions and a scoring system with each study having a potential score of 20: a study with 20 points was deemed high quality, 16–19 good quality and ≤ 15 low quality.

### 2.4 Data Synthesis

**Figure 1:**
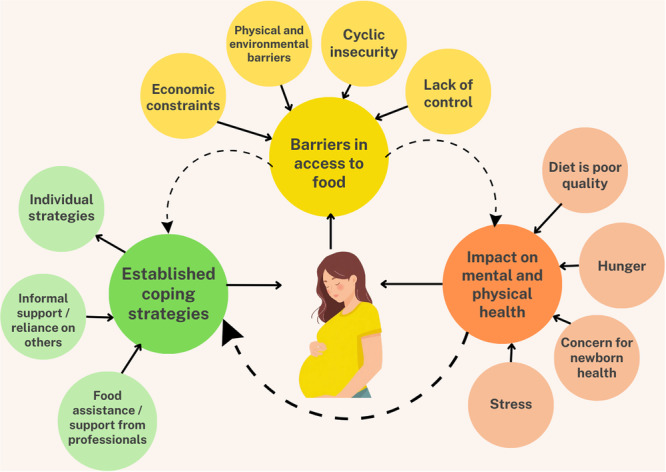
Visual map of themes identified by a meta‐synthesis of qualitative studies exploring pregnant women's experiences of food insecurity in high‐income countries (HICs).

A three‐stage approach to thematic synthesis as described by Thomas and Harden [38] was conducted by SS and LC and involved: (1) the coding of text ‘line‐by‐line’, (2) the development of ‘descriptive themes’ and (3) the generation of ‘analytical themes’. All studies were first uploaded to NVivo 14 and read in‐depth before open coding was applied to both authors' interpretations in the main body of the text, as well as the quotations used by authors to support their interpretations. The development of subsequent descriptive themes was aided by reflexive analysis meetings and hand drawing of visual thematic maps in order to link codes and group them into overarching themes. The final stage of coding involved the generation of analytical themes, which determined the key messages beyond descriptive themes [39]. At the end of this stage, coding and hand‐drawn thematic maps were combined to create a final visual summary of our themes (see Figure 1).

The research team consisted of eight authors, all female. Several had experienced pregnancy, and all reported food security during the study. One member of our team had historical experience of food insecurity, but not related to pregnancy or recent. With the exception of one author—who was a master's student during the study—all other authors reported working in maternal, nutrition and/or inequalities research spanning 5–20 years. These shared and differing experiences shaped data extraction, synthesis and interpretation. We acknowledge that the team's demographic profile, particularly our food‐secure status, contrasted with many participants included in our synthesised findings. Thus, during reflexive discussions, we took time and care to examine our positionalities, guard against our own assumptions and represent participants' perspectives authentically.

Finally, the GRADE‐CERQual approach was applied to assess confidence in each theme generated by this thematic synthesis, classified as high, moderate, low, or very low [40]. Confidence was assessed based on four key components: methodological limitations, coherence, data adequacy, and relevance. In the results that follow, italic text represents data extracted from the included studies verbatim. Double quotation marks were used for direct participant quotations from the included studies, and single quotation marks for authors' interpretations of data reported in the included studies.

## 3 Results

**Figure 2:**
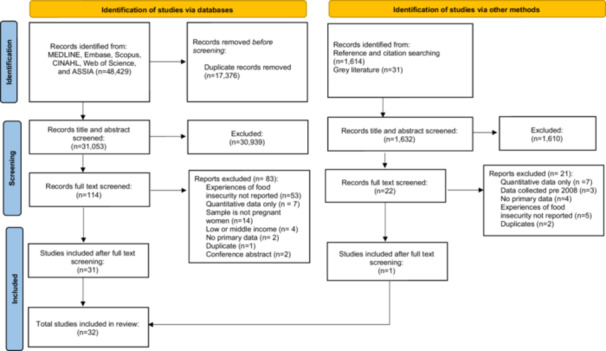
Flow diagram of the literature search and filtering results for a systematic review of pregnant women's experiences of food insecurity in high‐income countries (HICs) [34].

After de‐duplication, database searches identified 32,685 potentially eligible records, and 31 met the inclusion criteria after screening. An additional 22 potentially eligible studies were identified from citation chaining and grey literature searches, and one of these met the inclusion criteria. A total of 32 studies were included following all stages of searching (Figure 2).

Table 1 shows the characteristics of included studies and key findings organised according to synthesis themes and sub‐themes, whilst Supporting Information S1: Table S4 provides additional study characteristics. Food insecurity was explicitly discussed in 13 studies [41, 42, 43, 44, 45, 46, 47, 48, 49, 50, 51, 52, 53], and in the remaining 19 studies [54, 55, 56, 57, 58, 59, 60, 61, 62, 63, 64, 65, 66, 67, 68, 69, 70, 71, 72], food insecurity was implied in their reported data on women's experiences. Most studies were conducted in the United States of America (USA) (*n* = 15), followed by the United Kingdom (*n* = 7), Canada (*n* = 5), Australia (*n* = 2), Germany (*n* = 1), Denmark (*n* = 1) and France (*n* = 1). Included studies spanned various marginalised groups, including women in recovery [41], refugee and asylum‐seeking populations [59, 61], women experiencing homelessness or living in temporary accommodation [44, 56], Aboriginal and/or Torres Strait Islander communities [43], and women in detention centres [55]. Sample sizes ranged from 4 to 100, with a pooled sample of 693 women. A total of 23 studies (72%) reported participants' age, which ranged from 16 to 47 years. A total of 28 studies (88%) reported participant ethnicity, race or nationality, spanning but not limited to White, African American, Aboriginal, Hispanic and/or Latina, Somali, Nepali, Native American, Asian, Indian, West African, Pakistani, gypsy traveller, Bedoon, Caribbean and Puerto Rican.

**Table 1:** Characteristics and key themes of included studies exploring pregnant women's experiences of food insecurity in high‐income countries (HICs) (*n* = 32).

Author, year	Country	Study aim	Participant characteristics	Food insecurity explicit/implied	Key themes/sub‐themes
Allen et al. 2024 [41]	USA	Describe the unique social challenges faced by rural pregnant women with intersecting SUD^a^ and unmet social needs.	*n* = 4 rural women with food insecurity in recovery for SUD (*n* = 3 currently pregnant, *n* = 1 gave birth in the 15 weeks previously) *n* = 2 reported experiences of interpersonal violence; *n* = 2 disclosed histories of mental health challenges	Explicit	Negative physical wellbeing Transportation Reliance on ‘others’
Allen et al. 2023 [42]	USA	Describe the experience of food insecurity and food access among women in northern New England during the perinatal period	*n* = 14 (*n* = 12 currently pregnant, *n* = 2 gave birth up to 15 weeks previously)	Explicit	Income and employment Transportation Negative mental wellbeing Established coping strategies
Anderson et al. 2015 [54]	USA	Examine factors implicated in GWG^b^ in low‐income, overweight and obese women	*n* = 29 Ethnicity *n* = 14 black, *n* = 12 white non‐Hispanic, *n* = 3 Hispanic Parity *n* = 11 first pregnancy *n* = 12 first half of pregnancy; *n* = 10 last half of pregnancy; *n* = 7 post‐partum	Implied	Transportation Cost of food Diet is poor quality Lack of control
Arshad et al. 2018 [55]	United Kingdom	Explore pregnant migrant women's experiences of living in detention, in order to understand maternity care provision and the impact of detention on pregnant migrant women's health	*n* = 4 Ethnicity *n* = 2 Eastern Europe, *n* = 1 Sub‐Saharan, *n* = 1 Indian All first pregnancies, all up to 2 years post‐partum at point of interview	Implied	Hunger Diet is poor quality Lack of control
Booth et al. 2023 [43]	Australia	Determine Aboriginal and Torres Strait Islander parents’ perceptions and lived experiences of food insecurity and suggestions to improve food insecurity in remote communities	*n* = 17 All women from Aboriginal and/or Torres Strait Islander communities (eight women from Cape York, nine women from Central Australia)	Explicit	Transportation Reliance on others Cultural identity Negative mental wellbeing Income and employment Cyclic insecurity Hunger Established coping strategies
Borghi et al. 2023 [56]	France	Describe how health of homeless women who are pregnant or in the postpartum period is impacted, according to them, by their physical, social and healthcare surroundings	*n* = 26 (*n* = 10 pregnant, *n* = 10 postpartum period, *n* = 6 from social surroundings) Age pregnant women 21.0 (3.3); postpartum women 26.0 (5.0); members of social surroundings people 31.5 (15.3) Ethnicity *n* = 26 French Marital status pregnant women *n* = 4 single, *n* = 6 married; postpartum women *n* = 5 single, *n* = 5 married; members of social surroundings *n* = 6 married	Implied	Barriers in access Cost of food Negative physical wellbeing
Burton et al. 2024 [57]	USA	Explore determinants of food choices and dietary perspectives of healthy eating from young, urban Black pregnant women	*n* = 11 Age range 18 to 25 yo^c^ Race *n* = 9 Black women Ethnicity *n* = 9 non‐Hispanic Marital status *n* = 11 single Employment *n* = 6 full time, *n* = 3 not employed Education *n* = 7 high school, *n* = 2 some college Income range from US$520 to US$2250 per month or US$12.50 to US$16 per hour	Implied	Cost of food Diet is poor quality Barriers in access
Cumming and Symon. 2025 [44]	United Kingdom	Explore maternal experiences of pregnancy while living in temporary accommodation in the United Kingdom.	*n* = 14 women between 28 weeks pregnant and 24 months postnatal who identified as homeless during pregnancy Age range 19–40 yo (*n* = 2 not disclosed) Parity *n* = 5 primiparous; *n* = 9 2–6 children *n* = 2 antenatal; *n* = 12 postnatal Ethnicity *n* = 2 white British; *n* = 2 white Scottish; *n* = 1 gypsy traveller; *n* = 4 black African; *n* = 1 Eastern European; *n* = 1 Bedoon; *n* = 1 Pakistani; *n* = 1 not disclosed	Explicit	Negative physical and mental wellbeing Diet is poor quality Barriers in access Lack of control Hunger Cost of food Reliance on others Cultural identity
Dundas et al. 2023 [58]	United Kingdom	Understand the lived experiences of those intended to benefit from the Healthy Start Vouchers scheme, the processes involved in the take‐up or non‐take‐up of the vouchers, how the vouchers were used and perceived by women	*n* = 40 Age range 22–47 yo Ethnicity *n* = 24 White Scottish; *n* = 6 White British; *n* = 3 Black African; *n* = 2 British Pakistani; *n* = 1 Pakistani; *n* = 1 Nepalese; *n* = 1 White Australian; *n* = 1 White Irish; *n* = 1 White Polish Education *n* = 16 no qualifications; *n* = 8 high school; *n* = 6 college; *n* = 9 (studying) degree; *n* = 1 postgraduate degree Parity *n* = 9 one child; *n* = 23 two or three children; *n* = 8 ≥ 4 children	Implied	Cost of food Cyclic insecurity Established coping strategies
Ellul et al. 2020 [59]	United Kingdom	Explore vulnerable migrant women's lived experience of being pregnant and destitute in order to influence local and national policy and address these gaps	*n* = 6 All women originated from different countries in the African subcontinent	Implied	Negative mental wellbeing Hunger Reliance on others
Evenosky et al. 2021 [60]	USA	Evaluate how pregnant women living in a predominantly Black/Latino community with high preterm birth rates navigate their food environment	*n* = 7 *n* = 5 pregnant at time of interview; *n* = 2 gave birth 1–2 months prior to interview Age *n* = 2 20–29 years, *n* = 4 30–40 years and *n* = 1 over 40 years Ethnicity *n* = 2 non‐Hispanic black, *n* = 3 non‐Hispanic white, *n* = 2 non‐Hispanic black/white mixed Parity *n* = 2 first pregnancy, *n* = 1 one child and *n* = 4 multiple children Education *n* = 2 some high school, *n* = 2 high school graduate, *n* = 1 technical school or associates degree, *n* = 2 some college	Implied	Cost of food Transportation Diet is poor quality Cyclic insecurity Established coping strategies
Gewalt et al. 2019 [61]	Germany	Gain in‐depth insights and understanding into pregnant asylum seekers’ experiences and perceived needs whilst living in state‐provided accommodation in Germany	*n* = 9 Age between 22 and 37 yo Ethnicity *n* = 5 West African, *n* = 3 Eastern Europe, *n* = 1 South Asian Single parent *n* = 5 Education *n* = 5 less than high school, *n* = 2 grade school, *n* = 1 high school and *n* = 1 none Parity *n* = 5 first pregnancy	Implied	Diet is poor quality Hunger Lack of control Cultural identity
Graham et al. 2016 [45]	USA	Understand the association between socioecological factors and poor health weight outcomes in low‐income women and compare from pregnancy to post‐partum	*n* = 45 (*n* = 15 pregnant; *n* = 30 up to 18 months post‐partum) Age between 18 and 35 Ethnicity *n* = 34 African American, *n* = 8 Hispanic, *n* = 11 White Parity *n* = 18 pregnant with first child	Explicit	Transportation Cost of food Income and employment Hunger Diet is poor quality
Gross et al. 2019 [46]	USA	Understand practices in the context of food insecurity to better inform prevention interventions	*n* = 100 Age average of 30 Ethnicity Hispanic and/or Latina Primarily born outside the United States (87%) Spanish speaking (87%), *n* = 32 with 3–7‐month‐olds, *n* = 31 with 10–15‐month‐olds, and *n* = 37 with 19–24‐month‐olds. Living with married partner *n* = 76 Educational attainment less than high school (41%)	Explicit	Diet is poor quality Established coping strategies Cyclic insecurity Income and employment
Groth et al. 2016 [62]	USA	Understand how factors (e.g., cravings, taste, appetite) affect dietary decision making, and to determine the barriers to adoption of a healthy diet during pregnancy among African American women who are low income and reside in an urban area.	*n* = 25 Age: mean 25 yo (5.6) Gestation week: 10–20 (*n* = 3), 21–20 (*n* = 11), 31–40 (*n* = 11) Parity *n* = 6 nulliparous; *n* = 19 multiparous Employment *n* = 9 employed	Implied	Barriers in access Cost of food Concern for newborn health
Hromi‐Fiedler et al. 2016 [63]	USA	Explore the prenatal factors that promote and hinder fruit and vegetable intake in pregnant women	*n* = 45 Age: average of 24.5 Average of 19 weeks gestation at recruitment. Ethnicity *n* = 21 Puerto Rican Employed *n* = 10 Single parent *n* = 18 Education *n* = 23 had at least high school Parity *n* = 11 first pregnancy	Implied	Cost of food Cyclic insecurity Diet is poor quality Transportation
Iqbal et al. 2024 [64]	United Kingdom	Explore the health concerns of pregnant Pakistani women living in deprived inner‐city areas of Bradford	*n* = 21 Recruited between 26 and 28 weeks gestation	Implied	Barriers in access Cost of food Cultural identity
Marshall et al. 2026 [47]	United Kingdom	Explore food insecurity experiences during pregnancy and postpartum among a multi‐ethnic group and identify necessary support.	*n* = 11 mean age 33 ± 14 years currently pregnant (*n* = 2); infant under 12 months (*n* = 9) Black African/Caribbean/Black British *n* = 9; white *n* = 2 Household income: below £19,000 (*n* = 8); £19,000 to £31,999 (*n* = 1); £32,000 to £63,999 (*n* = 2) Education *n* = 2 secondary school; *n* = 2 professional training; *n* = 6 undergraduate/nursing qualification; *n* = 1 postgraduate degree Parity: *n* = 1 0/pregnant with first; *n* = 4 1 child; *n* = 6 2+ children Housing: renting privately (*n* = 4); renting from Housing Association (*n* = 7)	Explicit	Cost of food Negative physical and mental wellbeing Stress Diet is poor quality Established coping mechanisms Barriers in access Transport Income and employment Reliance on others
McKerracher et al. 2020 [65]	Canada	To support pregnancy nutrition and reduce dietary inequalities by investigating what influences diet in pregnancy and how people can be supported to improve diets when pregnant	*n* = 22 Age between 21 and 44 yo Ethnicity *n* = 10 native English speakers and *n* = 12 non‐native English speakers Parity ranged from 0 to 3 children Pregnant *n* = 9; postpartum *n* = 13	Implied	Cost of food Diet is poor quality Income and employment Reliance on others
Nagourney et al. 2019 [66]	USA	Explore attitudes and perceptions surrounding diet and exercise among low‐income obese African‐American pregnant women in Baltimore	*n* = 21 Age ranging from 18 to 33 All enroled in the first or second trimester Ethnicity African American Education *n* = 5 did not complete high school, *n* = 9 had general education development or diploma, *n* = 6 had some college but no college degree Single parent *n* = 19 unmarried, *n* = 2 married	Implied	Cost of food
Ohly et al. 2018 [67]	United Kingdom	Develop in‐depth, evidence‐based explanations for how low‐income pregnant women use Healthy Start vouchers and explore outcomes of the programme and develop explanations for why these might occur	*n* = 11 Age *n* = 7 18–35 yo and *n* = 4 26–35 yo Ethnicity *n* = 11 white British Single parent *n* = 6 Parity *n* = 2 first pregnancy and *n* = 9 multiple children *n* = 5 pregnant at time of interview; *n* = 6 pregnant within previous 6 months	Implied	Reliance on others Hunger Diet is poor quality
Oresnik et al. 2025 [48]	Canada	To understand, from a syndemic perspective, the intersections among food insecurity, GDM^d^ and anxiety/mood disorders during pregnancy in Canada.	*n* = 28 (focus groups *n* = 22; interviews *n* = 6) Focus groups: Age between 21 and 44 yo Ethnicity *n* = 10 native English speakers and *n* = 12 non‐native English speakers Parity ranged from 0 to 3 children Pregnant *n* = 9; postpartum *n* = 13 Interviews: Age between 25 and 34 yo Parity ranged from 0 to 3 children Occupation: *n* = 4 full time; *n* = 1 part‐time; *n* = 1 stay‐at‐home parent Birth country: *n* = 4 Canada; *n* = 1 Iran; *n* = 1 Romania Relationship: *n* = 4 married; *n* = 1 single but co‐living; *n* = 1 separated	Explicit	Cost of food Diet is poor quality Negative physical and mental wellbeing Stress Barriers in access Transport Income and employment Reliance on others
Paul et al. 2013 [68]	USA	Gain an understanding of issues related to GWG including general health, diet and physical activity among high‐ and low‐income women and to elucidate socio‐ecological and psychosocial risk factors that increase risk for excessive GWG	*n* = 15 (low‐income) Age 18–35 Ethnicity *n* = 11 African American, *n* = 1 Asian *n* = 3 Hispanic, *n* = 14 White Mean weeks gestation 25.2 Parity *n* = 16 first pregnancy, *n* = 2 one child, *n* = 8 more than one child	Implied	Lack of control Concern for newborn health Diet poor quality
Quintanilha et al. 2018 [49]	Canada	Explore experiences in coping with household food insecurity	*n* = 17 Somali women Single parent *n* = 6 0–3 children (*n* = 9), 4–6 children (*n* = 5), 7 or more children (*n* = 3) At least one adult in household employed (*n* = 7), social assistance (*n* = 10)	Explicit	Diet is poor quality Lack of control Stress Cultural identity
Reyes et al. 2013 [69]	USA	Understand the perceptions of low‐income, overweight, and obese, African American mothers about diet quality in pregnancy with a focus on the barriers and facilitators of healthy eating	*n* = 21 Age average 23.8 yo; between 18 and 37 yo Ethnicity *n* = 21 African American Single parent *n* = 19 single but all living with adults or children Employment *n* = 10 unemployed Education *n* = 7 not completed high school Majority in their first or second trimester of pregnancy (*n* = 15) Parity *n* = 16 multiparous	Implied	Cost of food Transportation Diet is poor quality Cyclic insecurity Hunger Established coping mechanisms
Sim et al. 2020 [50]	Canada	Examine how participants experienced breastfeeding in context of food insecurity and high body weight	*n* = 8 Majority identified as Caucasian Participants interviewed at three time points: after their first trimester of pregnancy (*n* = 8), 1 month after giving birth (*n* = 6), and 3 months after giving birth (*n* = 6).	Explicit	Negative mental wellbeing Worry about newborn health
Struthers et al. 2019 [70]	Canada	Describe how an unconditional cash transfer programme, the HBPB^e^, improved the birth outcomes among low‐income mothers and how it narrowed the equity gap in birth outcomes between high‐ and low‐income families	*n* = 20 Age between 18 and 40 years Parity *n* = 4 first pregnancy, *n* = 5 had children who were not living with them	Implied	Negative mental wellbeing Reliance on others Cost of food
Waberi et al. 2025 [51]	Denmark	Explore how household food insecurity is experienced in relation to health and various other social and emotional challenges for people living in neighbourhoods expected to have relatively high rates of household food insecurity during the perinatal periods	*n* = 5 (1 pregnant, 4 postpartum) Age between 20 and 44 years Average no of children: 2.6	Explicit	Income and employment Cost of food Diet is poor quality Negative physical and mental wellbeing Hunger Stress Reliance on others Lack of control Cultural identity
Wise et al. 2015 [71]	USA	Examine beliefs about healthy eating, barriers to healthy eating, and nutrition education preferences of pregnant adolescents	*n* = 14 Age *n* = 6 aged 16, *n* = 5 aged 17, *n* = 1 aged 18 and *n* = 2 aged 19 Ethnicity *n* = 11 Hispanic, *n* = 2 Black and *n* = 1 white Education *n* = 14 high school students Living with parents (*n* = 12), living with boyfriend's parents (*n* = 1), living in home for single mothers (*n* = 1) Gestation week: up to 12 (*n* = 6), 13–24 weeks (*n* = 5), 25 weeks to term (*n* = 3)	Implied	Cost of food Transportation Lack of control
Whisner et al. 2016 [72]	USA	Assess the general eating behaviours and beliefs of a primarily African American cohort of pregnant adolescents	*n* = 66 Age average of 16.8 yo Ethnicity *n* = 12 Hispanic and *n* = 54 non‐Hispanic Living with parent (*n* = 44), living alone (*n* = 2), other living arrangements (*n* = 20) Parity *n* = 8 had a parity greater than 1	Implied	Reliance on others Cost of food
Yee et al. 2016 [52]	USA	Investigate nutrition‐related challenges of diabetes during pregnancy in low‐income minority pregnant women	*n* = 10 Age average of 28.5 Ethnicity *n* = 4 African American, *n* = 4 Latina, n = 1 Native American, *n* = 1 Asian Marital status *n* = 3 single, *n* = 1 married, *n* = 5 significant other and *n* = 1 other Parity *n* = 8 multiparous	Explicit	Lack of control Cost of food Cyclic insecurity Income and employment
Zinga et al. 2022 [53]	Australia	Determine Australian food‐insecure pregnant women's experiences and perceptions of healthy eating in pregnancy	*n* = 7 Age between 25 and 41 Ethnicity *n* = 5 Australian born, *n* = 1 Indian born and *n* = 1 African born None of sample employed Parity *n* = 4 first pregnancy, *n* = 2 second pregnancy, *n* = 1 pregnant with their sixth child Gestational age ranged from 12 to 36 weeks	Explicit	Cost of food Diet is poor quality Established coping strategies

^a^ SUD = substance use disorder.

^b^ GWG = gestational weight gain.

^c^ yo = years old.

^d^ GDM = gestational diabetes mellitus.

^e^ HBPB = healthy baby prenatal benefit.

Data from both pregnant and postpartum women were combined in 13 studies, seven studies reported data from pregnant women only, two studies reported data from postpartum women only, and in the remaining 10 studies, this was unclear. Most studies (*n* = 23) were deemed good quality [41, 43, 44, 45, 46, 47, 48, 49, 50, 52, 54, 57, 58, 59, 60, 62, 63, 64, 65, 66, 68, 69, 71], six were rated high quality [42, 53, 56, 61, 67, 70] and three low quality [51, 55, 72] (Table 2). For good and low‐quality studies, reflexivity was often not explicitly discussed in the methodology. Meanwhile, ethical issues were frequently mentioned only in reference to a statement of approval from an ethics committee. In other words, ethics was predominantly reported procedurally (rather than relationally) with little included details on ethical decision making, tensions or challenges ‘in the field’. All studies had a clear and relevant aim, and most justified that the use of a qualitative methodology was appropriate. Specific research design, methods used and recruitment strategy were also mainly appropriate, but for some studies deemed good or low quality, explicit justification was lacking.

**Table 2:** Results of quality assessment of studies exploring pregnant women's experiences of food insecurity in high‐income countries (HICs) using the Critical Appraisal Skills Programme (CASP) Qualitative Studies Checklist^a^, ordered alphabetically.

Study	Clear relevant aim?	Appropriate methodology?	Appropriate research design?	Appropriate recruitment strategy?	Appropriate data collection method?	Reflexivity discussed?	Ethical issues been considered?	Sufficiently rigorous analysis methods?	Clear statement of finding?	Valuable research question?	Score* quality
Allen et al., 2024 [41], **USA**	Yes	Yes	Yes	Yes	Yes	Yes	Can't tell	Yes	Yes	Yes	19 Good
Allen et al. 2023 [42], **USA**	Yes	Yes	Yes	Yes	Yes	Yes	Yes	Yes	Yes	Yes	20 High
Anderson, 2015 [54], **USA**	Yes	Yes	Yes	Yes	Can't tell	Can't tell	Can't tell	Can't tell	Yes	Yes	17 Good
Arshad, 2018 [55], **United Kingdom**	Yes	Yes	Yes	Can't tell	Yes	No	Can't tell	Can't tell	Can't tell	Can't tell	13 Low
Booth, 2023 [43], **Australia**	Yes	Yes	Yes	Yes	Yes	Yes	Yes	Yes	Can't tell	Yes	19 Good
Borghi et al. 2023 [56], **France**	Yes	Yes	Yes	Yes	Yes	Yes	Yes	Yes	Yes	Yes	20 High
Burton et al. 2024 [57], **USA**	Yes	Yes	Yes	Yes	Yes	Can't tell	Yes	Yes	Yes	Yes	19 Good
Cumming and Symon, 2025 [44], **United Kingdom**	Yes	Yes	Yes	Yes	Yes	Can't tell	Yes	Yes	Yes	Yes	19 Good
Dundas et al. 2023 [58], **United Kingdom**	Yes	Yes	Yes	Yes	Yes	No	Can't tell	Can't tell	Yes	Yes	16 Good
Ellul, 2020 [59], **United Kingdom**	Yes	Yes	Yes	Yes	Yes	Can't tell	Can't tell	Can't tell	Yes	Yes	18 Good
Evenosky, 2021 [60], **USA**	Yes	Yes	Yes	Yes	Yes	No	Yes	Can't tell	Yes	Yes	17 Good
Gewalt, 2019 [61], **Germany**	Yes	Yes	Yes	Yes	Yes	Yes	Yes	Yes	Yes	Yes	20 High
Graham, 2016 [45], **USA**	Yes	Yes	No	Yes	Can't tell	Can't tell	Yes	Yes	Yes	Yes	16 Good
Gross, 2019 [46], **USA**	Yes	Yes	Can't tell	Yes	Yes	Can't tell	Can't tell	Yes	Yes	Yes	17 Good
Groth et al. 2016 [62], **USA**	Yes	Yes	Yes	Yes	Yes	No	Yes	Yes	Yes	Yes	18 Good
Hromi‐Fiedler, 2016 [63], **USA**	Yes	Yes	Yes	Yes	Yes	Can't tell	Can't tell	Yes	Yes	Yes	19 Good
Iqbal et al. 2024 [64], **United Kingdom**	Yes	Yes	Yes	Yes	Yes	Can't tell	Yes	Yes	Yes	Yes	19 Good
Marshall, 2026 [47], **United Kingdom**	Yes	Yes	Yes	Yes	Yes	Yes	Can't tell	Yes	Yes	Yes	19 Good
McKerracher, 2020 [65], **Canada**	Yes	Yes	Can't tell	Can't tell	Yes	Can't tell	Can't tell	Yes	Yes	Yes	16 Good
Nagourney, 2019 [66], **USA**	Yes	Yes	No	Yes	Yes	No	No	Yes	Yes	Yes	16 Good
Ohly, 2018 [67], **United Kingdom**	Yes	Yes	Yes	Yes	Yes	Yes	Yes	Yes	Yes	Yes	20 High
Oresnik et al., 2025 [48], **Canada**	Yes	Yes	Yes	Can't tell	Yes	No	Can't tell	Yes	Yes	Yes	16 Good
Paul, 2013 [68], **USA**	Yes	Yes	Yes	Can't tell	Can't tell	Can't tell	Can't tell	Yes	Yes	Yes	16 Good
Quintanilha 2018 [49], **Canada**	Yes	Yes	Can't tell	Yes	Yes	Can't tell	Yes	Yes	Yes	Yes	19 Good
Reyes, 2013 [69], **USA**	Yes	Yes	No	Yes	Yes	Yes	Yes	Yes	Yes	Yes	18 Good
Sim, 2020 [50], **Canada**	Yes	Yes	Yes	Yes	Yes	Yes	Yes	Yes	Can't tell	Yes	19 Good
Struthers, 2019 [70], **Canada**	Yes	Yes	Yes	Yes	Yes	Yes	Yes	Yes	Yes	Yes	20 High
Waberi et al., 2025 [51], **Denmark**	Yes	No	Yes	Yes	Yes	Yes	Can't tell	No	Yes	Yes	15 Low
Wise, 2015 [71], **USA**	Yes	Yes	Yes	Yes	Yes	Can't tell	Can't tell	Yes	Yes	Yes	19 Good
Whisner, 2016 [72], **USA**	Yes	Can't tell	Can't tell	Can't tell	Yes	Can't tell	Can't tell	Yes	Yes	Yes	15 Low
Yee, 2016 [52], **USA**	Yes	Yes	Yes	Yes	Yes	Can't tell	Yes	Yes	Yes	Yes	19 Good
Zinga, 2022 [53], **Australia**	Yes	Yes	Yes	Yes	Yes	Yes	Yes	Yes	Yes	Yes	20 High

^a^ The checklist is made up of 10 questions and a scoring system with each study having a potential score of 20; studies with 20 points were deemed high quality, 16–19 good quality and ≤ 15 low quality.

Three overarching analytical themes were identified across all included studies: (1) barriers in access to food; (2) impact on physical and mental health; and (3) established coping strategies. As well as being unpacked in the sub‐sections which follow, themes and sub‐themes are also presented as a visual summary in Figure 1. All three themes had a similar underlying thread relating to women's experiences of food insecurity outside of pregnancy, appearing to be exacerbated during pregnancy. The GRADE‐CERQual assessment yielded moderate to high confidence ratings for all three analytical themes. Overall assessments are presented in Supporting Information S1: Table S5 alongside of additional empirical data across included studies to further contextualise all themes and sub‐themes.

### 3.1 Theme One: Barriers in Food Access

Economic, physical, and environmental barriers were paramount in women's access to food. These barriers did not act in silo and interacted for women experiencing food insecurity during pregnancy. Pregnant women frequently described how a lack of money dictated their food choices: “*eighty‐percent of the fear when you first found out you're pregnant is, how am I going to do this? How am I going to afford this?”* [54]. The cost of fresh produce, including fruit, vegetables, milk, and poultry, was deemed to be particularly expensive and limited to occasional purchases. Consequently, diets were not based on what pregnant women wanted to eat, but what was most accessible and affordable to try and reduce hunger for themselves and their household. With a limited budget, women were eager to waste minimal food and so opted for longer shelf life and easy storage foods, *“looking at the budget and what we have, either I get that stalk of celery or I could get those four boxes of pasta that I could have dinner for a couple more days”* [52]. Fluctuating food supply and nutritional quality were linked to lack of employment opportunities, scarcity of childcare and the reduced or cyclical nature of income, the latter of which meant that the severity of food insecurity tended to increase towards the end of the month as money or food vouchers dwindled “*When I got pregnant, the issues began with having food…. I had to quit my job because I couldn't stand standing on my feet for very long*” [42]. Meanwhile, employed women described working multiple jobs, limited employer flexibility, and balancing work with caregiving responsibilities, all of which led to difficulty prioritising their own nutrition and meal timings. For other populations, automatic income deductions, such as government penalties, debt repayments or personal loans, resulted in ‘short pay’ and varied the amount of money available per week ‘*to do proper shopping for food’* [43].

Economic barriers were particularly influential on the pregnancy diets of marginalised groups such as migrant women or those living in remote areas (e.g., Aboriginal and/or Torres Strait Islander communities) [43]. For example, cost was a barrier to purchasing and consuming traditional foods associated with women's home countries or with religious meaning, such as Halal meat. The unaffordability of these foods led to a further loss of cultural identity [49]. Further, living in temporary accommodation impacted the diets of those homeless due to the lack of cooking and storage facilities which meant women had to relinquish control and either relied on friends (and cooked elsewhere), skipped meals or had access to bare minimal equipment such as a kettle in their bedroom or shared kitchen space with multiple residents: *“The 115 (the emergency accommodation number) has put us in a place where I cannot cook, so I must go and cook at somebody else's house […] If I want to eat, I must go out. […] It's complicated to go to somebody else's house all the time.”* [56]. Such lack of control and regular food insecurity left women living in temporary accommodation feeling undermined and eroded their sense of being “good” mothers: “*It just makes you feel really low… it made me feel like a bad parent because I couldn't provide for my child*” [44].

Whilst qualitative data extracted precluded any direct pregnancy versus pre‐pregnancy comparison, pregnancy appeared to heighten the challenges that women already faced relating to transport, creating physical barriers to accessing (healthy) food. Across included studies, there were low levels of car ownership; many women were constrained to either walking or using public transport. Walking introduced additional environmental barriers to accessing healthy foods, as walking led to eating at fast‐food restaurants, or shopping at local stores, which tended to be more expensive and have limited healthy food choices and greater high‐fat, high‐sugar, high‐salt options. Pregnancy symptoms exacerbated barriers to food shopping as transport became more difficult. For example, when pregnant women relied on walking as their mode of transport, they reported *‘choosing a closer store over a further one due to pregnancy‐related fatigue’* [60] and “*sometimes you don't have a way for you to get to the store for stuff for a healthy meal and so you just have to eat what is close to you”* [45]. To overcome these barriers, some women were dependent on family, friends, or neighbours to access food: *“I like to go to a supermarket. But I mean, I can get there, I just got to wait for when it's convenient for someone to take me, which is hard”* [63]. Established coping mechanisms, including support such as this from informal networks, are unpacked further across theme three below. Meanwhile, others used taxis, creating additional economic costs to an already limited household budget: *“You have to pay someone* (US) *$10 or* (US) *$15 to take you to the grocery store, or a cab, and that's expensive”* [54].

### 3.2 Theme Two: Impacts on Physical and Mental Health

The barriers to accessing food had implications on pregnant women's physical and mental health, including concerns over maternal diet quality, maternal hunger, maternal well‐being and newborn health, described by Oresnik et al. [48] as a ‘syndemic’, specifically “*multiple, multi‐direction pathways of association between anxiety and mood disorder, food insecurity and income‐related challenges during pregnancy*”. Pregnant women frequently described the feeling of compromising diet quality, which brought about feelings of frustration, stress, anxiety and concerns about the impact of poor diet quality on their developing baby. Meanwhile, food‐insecure women also expressed difficulty maintaining a healthy weight in pregnancy, where women “*consumed adequate calories for their pregnancy but were dissatisfied with the nutritional quality of their meals*” [53].   Further, a negative body image was frequently discussed in relation to weight gain from eating food deemed to be nutritionally poor: “*I never seem to have enough good food. The food that I have is crap… therefore, I feel like crap and, therefore, I look like crap”* [50].

Pregnant women described regularly experiencing feelings of hunger from ‘*decreasing size and frequency of meals’* [49] and ‘*not eating for days at a time’* [45], which would sometimes present as physical pain and emotional distress: “*You just ignore all of the alarms ringing in your body. And then, all of a sudden, my body just couldn't anymore. It started shaking and I was like ‘auughhhhh’ and was thinking ‘what the hell is going on here… why do I always have headaches… why am I crying without a reason?’. I Thought I was just stressed because of work [but I was sick and pregnant]. And then finally it's your body that takes over and tells you how to be*” [51]. Food rationing over sustained periods of time normalised the feeling of hunger for some women: who *“…could probably go most of the day without eating and that wouldn't affect me too much. I mean, obviously it does, but not hunger‐wise”* [53]. The normalisation of hunger was also particularly apparent for pregnant women in detention who did not have control over when, where, and what they could eat: ‘*all the women felt hungry in between meals, especially in the evenings and were not allowed to take any food back to their room to snack’* [55]. Meanwhile, feeling physically unsafe and vulnerable meant that pregnant women's health and wellbeing (including adequate nutrition) whilst living in temporary accommodation due to homelessness and/or migration status could not be prioritised.

Women were concerned that their newborn would not receive sufficient nutrients due to their poor diet quality, inducing feelings of guilt and impacting maternal mental wellbeing. Concerns extended to *‘fears that their own poor diet would adversely affect breast milk quantity and quality’* [46]. For some, their concerns about being unable to sustain breastfeeding or provide sufficient nutrients led to active decisions to limit breastfeeding duration or prefer to rely on formula as “*the only way he's (baby) getting any nutritional value”* [50]. Concerns about newborn health exacerbated maternal feelings of stress, and the burden of food insecurity on pregnant women created *‘mental fatigue’* with one participant describing how their “*head is throbbing, you're stressed all the time”* [53] and another recounting “*I was eating a little but not that much. That's why, when I have the baby, she is very small; she was so tiny*” [44].

Fuel poverty—or electricity scarcity—was a common stressor and appeared to operate in tandem with food insecurity for some pregnant women, leading to ‘trade‐offs’ in material needs: “*There's times where like okay, well, am I getting groceries for two weeks or am I paying hydro [electricity]*?” [48]. For example, electricity was needed to operate the fridge and freezer to ensure fresh food did not spoil, and other electricity use—such as air conditioners—was closely monitored so as not to significantly impact electricity credit available for food storage and cooking [43]. Meanwhile, stress amongst pregnant women also arose from worrying about feeding the other children in their family. Thus, women reported constantly thinking about food, planning meals, and where they were getting their next family meal from “*you're always thinking about what you are going to do and how you are going to do it”* [49]. Pregnancy created additional worry for the future and having sufficient food to feed *‘an extra mouth’*, in anticipation of infant formula costs, and new additional infant expenses that *‘forced families to choose between diapers and paying bills’* [46].

### 3.3 Theme Three: Established Coping Strategies

Across all included studies, some degree of food insecurity was normalised and accepted as part of pregnant women's everyday life. Women reported reliance upon varying degrees of individual‐level, familial‐level, community‐level, healthcare and societal strategies or programmes to ensure they were able to feed themselves and their household. On an individual level, women reported sacrificing other things to prioritise money for food, thus rationing food portions and ‘child first’ practices were well established across included studies to ensure children could eat first: “*us adults we don't really mind not having food, long as the kids get fed”* [43]. Pregnancy did not deter these rationing practices, even with the concern of newborn health, women continued to ensure children were fed first. Other individual coping strategies described across studies included planning ahead to avoid waste: “*My biggest thing is: I budget!”* [65]; staying hydrated with water and/or tea when food was unavailable; coupon clipping; use of personal judgement in sell‐by dates; creativity in use/substitution of ingredients; sourcing of ‘yellow sticker’ produce; and shopping late at night for discounts: “*I used to waste a lot of food that way, because it was like, “Oh, no, it's out of date, don't eat it.” it's not out of date, it's actually in good condition. So, yes … looking at the fruit or the veg, or the food in general, and going off of how it looks, rather than the dates*” [47]. In Booth et al. [43] exclusively, women outlined more diverse coping practices, including selling traditional items/food, gambling, synchronising shopping with others to buy in bulk, co‐ordinating with others according to pay cycles and utilising natural ‘Bush food’: “*everything out on country, like the bush food is all free*”.

External coping strategies extended to informal reliance on others for help in acquiring food. *‘Others’* varied from wider family members and friends to neighbours. Outside support from family, friends and neighbours was crucial to help mitigate the impact of food insecurity and ensure existing children had enough food in times when women ran out of food: *“Yeah, we did [run out of food]. A couple of times we did, and what would happen would, you know, he'll just, we'll borrow from his dad or stuff like that”* [52]. Family members and friendship groups formed valued social support networks for pregnant women when experiencing food insecurity, providing *‘safety nets’* [46] for women to loan money or have food provided [41]: “…*when we don't have enough food I …go ask around family members. …. I go to my first friend I ask her for. meat and bread and my friend will give me. I'll go to my second friend, I'll ask for cordial and then I go to my third friend, I will ask for noodle …. I usually go to couple of houses and return home with food…. … that cycle is really good Aboriginal cycle …food wise*” [43]. Such requests were rarely refused but—at times—caused those providing to run short themselves.

Finally, pregnant women across included studies outlined differing levels of structural care and support spanning government food assistance programmes, broader healthcare professionals and community‐level support structures. For some, healthcare professionals signposted to assistance which made it more accessible than the information being online: *“So, everybody at [the hospital] is literally superheroes right now to me because I wouldn't be able to do anything about the situation I'm in without them.”* [42]. Assistance received from benefit programmes was described as *‘critical’* with some women expressing beliefs that their food insecurity would be ‘*unmanageable*’ without such services: *“Thank God I always have enough money for food because I took government aid for my two children…that helps me a lot”* [46]. Studies set within the USA acknowledged both the Special Supplemental Nutrition Program for Women, Infants, and Children (WIC) and Supplemental Nutrition Assistance Program (SNAP) for providing support, whilst UK studies were focused on Healthy Start vouchers or localised Alexandra Rose Vouchers (ARV): “… *the lady from the Children's Centre [referred me to the Rose Vouchers], because I came in one day and was struggling a lot … she introduced me to the Rose Vouchers … I'm just really grateful for her, to step in and help with that*” [47]. Women spoke about the positive experiences they had from receiving food stamps or vouchers, which provided relief and allowed them to subsidise the higher costs of fresh fruit, vegetables, and meat. Having food vouchers gave women greater freedom over the foods they were able to buy without choices being limited solely to cost: “*Now that I have the vouchers, I can afford the extra fruits and vegetables without having to worry about the cost”* [67].

However, not all women had positive experiences with food assistance programmes, schemes or interventions and pregnant women outlined a number of barriers and/or inhibiting factors such as vouchers not stretching far enough, rejection/ineligibility, shame/stigma, lack of awareness or ‘capital’ needed to negotiate benefits systems and the perception that others need this help more: “*… it's embarrassing. Everybody else is going through the same thing … I class myself as an independent adult and that's the last thing I want to discuss with anyone*” [47]. Meanwhile, marginalised groups such as migrant women, women in recovery, very low‐income communities and women living with homelessness experienced further barriers to accessing support from food assistance programmes and healthcare professionals: *“I've tried to apply for foods stamps, but because I don't have a stable address, they can't help me”* [46]. In places marginalised pregnant women did outline some positive examples of care (particularly where care was tailored, adapted or offered continuity), however,—predominantly—constraints in their living and personal circumstances led to them feeling judged, ‘unseen’ by professionals or to experience racism and discrimination: “*it's really nice that it [food support] even exists because people in situations like me [recovery from substance use dependency]… we'd just be going hungry completely”* [41]. Complexities within WIC policies specifically were further highlighted in one study where barriers were reported in relation to rules and regulations of what was and was not covered, *‘Hoops, jumping through hoops [referring to all the rules]’* [54]. Gross et al. [41] also found that whilst federal benefits may attenuate the severity of food insecurity women experience, it did not eliminate it, and that social stigma was another negative experience that some women saw as a barrier to using food vouchers with feelings of frustration and embarrassment associated with use in public, *“my face will get hot… because you get in line… because of the new changes, grocery stores haven't caught up with it yet”* [54].

Population‐level food assistance programmes were not referred to within research findings from other included studies spanning Australia, Germany, France, Denmark and Canada. Australia lacks a population‐level nutrition assistance programme (like WIC or Healthy Start) to support food‐insecure pregnant women. Thus, Australian women experiencing food insecurity during pregnancy currently need to navigate the food aid system themselves to arrange food assistance. Meanwhile, Canada does not have a single, population‐level food stamp programme similar to the US SNAP programme. Instead, food insecurity is largely addressed through a mix of targeted federal tax benefits, specific nutrition programmes, and a heavily used, non‐profit food bank network. Likewise, food assistance in Germany and France is currently provided through a combination of food bank/food aid initiatives, social welfare benefits and government‐backed health initiatives, with the French parliament—at the time of writing—debating the introduction of a proposed €150 monthly “food card”. Finally, in a departure from the above initiatives, all adult residents of Denmark pay significant income taxes, and these resources are then redistributed among families/households through a combination of services, subsidies and direct monetary transfers. This reflects historically low levels of household food insecurity across Denmark—nevertheless, these levels have risen in recent years and with broadly low levels potentially masking food insecurity experiences in marginalised populations [51].

## 4 Discussion

This systematic review is the first to synthesise qualitative data about pregnant women's experiences of food insecurity, with the aim to deepen understanding about the nuance and complexity that food insecurity imparts on this priority population group. Our review highlighted three interlinking themes, encompassing the impact of economic, physical and environmental barriers that prohibited optimal antenatal nutrition, the resultant physical and mental health concerns that women held for themselves and their baby, and their necessity to establish coping mechanisms. Findings from this review demonstrate that pregnancy appeared to heighten women's experiences of food insecurity and adversely impacted the reported physical and mental health of women. This reiterates the seriousness of food insecurity in pregnancy from the views of the women affected and further reiterates the urgency in action towards alleviating this serious and widespread public health issue.

Findings indicated that most women were intrinsically motivated to eat well during pregnancy. However, economic constraints were a stronger influence on food choices and food consumption. These findings concur with a 2003 report suggesting that women were aware of the components of a healthy diet, but ‘*without sufficient cash, no amount of nutrition information and knowledge will help to improve these women's diets*’ [73]. Food prices have been shown in several studies to be a key socio‐economic determinant of food choice for non‐pregnant food‐insecure adults [74, 75]. Food‐insecure individuals face simultaneous and competing influences when making food choices and are often forced to choose between buying food and paying bills, such as for housing and utilities, leaving food as a discretionary item [76]. This review suggests that this difficult but pragmatic process of decision‐making continues throughout pregnancy, despite the desire of pregnant women to eat well for their baby. Long term sustainability of proposed strategies targeting food insecurity in pregnancy must be carefully considered across settings which provide support to women during pregnancy and the postpartum period.

The finding that the cost of food overrides healthy food choices is particularly concerning, considering the importance of diet quality in supporting optimal maternal and child health outcomes. Adherence to healthy dietary patterns in pregnancy is associated with improved maternal health, optimal gestational weight gain and reduced risk of pregnancy complications [77, 78, 79] and supports the health and adequate growth of the fetus. Specifically, adequate consumption of fruit and vegetables and whole grains during pregnancy has been shown to reduce risk for multiple adverse birth outcomes [80], whilst facilitating intake of dietary fibre towards recommendations, has also been shown to benefit gut microbiome diversity, maternal glucose tolerance and healthy gestational weight gain [80]. This highlights the need to implement strategies during pregnancy that not only alleviate food insecurity but also promote healthy diet quality and optimal intakes of key nutrients and food groups, which benefit the health of both the mother and the developing baby.

This review identified that food‐insecure pregnant women experienced multiple stressors relating to their own access to food, their wider household, concerns about the impact of their diet on their unborn baby and worries about additional costs after the baby was born. The cyclic nature of food insecurity throughout the month appeared to exacerbate stress levels and the emotional labour or burden women tended to face in looking after the wellbeing (including nutrition) of their families, the latter of which is well established in extant literature [81, 82]. A recent meta‐ethnography demonstrated the embodiment of food insecurity for women, where food insecurity drew on all aspects of a woman's life [16]. This embodiment of food insecurity was also apparent for the pregnant women in this review, and its impact created feelings of stress. Stress is repeatedly associated with food insecurity in the literature, along with mental health conditions such as anxiety and depression [14]. Stress during pregnancy can have serious implications for both the mother's and the baby's health. Stress‐induced pregnancy complications amount to a significant cause of maternal and perinatal morbidity and mortality [83]. Research shows significant associations between stress, low‐birth weight and preterm birth and is related to slower cognitive development in the infant as a result of fetal exposure to stress hormones [84, 85]. One mechanism explains that during times of stress, glucocorticoids in the mother are released and enter fetal circulation, where they gain access to the fetal developing nervous system, potentially altering hippocampal development and impacting upon learning and memory [84, 86].

Across studies, women needed to establish coping strategies to ensure their household food supply. This supports the idea of food insecurity being a ‘managed process’ whereby women will strategise to avoid hunger [87, 88]. This review showed that women recalled strategies ranging from rationing more expensive items to pre‐planning shopping lists within a fixed budget. Reliance on family and friends was another key strategy women used to mitigate the impact of food insecurity during pregnancy. The reliance on informal networks to ensure food needs are met is well documented in female‐headed households [89, 90]. Previous research has shown that women ‘work hard to prevent hunger amongst their children’ and their adaptation of various strategies is key to prioritising their children's needs [88]. Further, being pregnant does not change this approach despite higher maternal nutritional needs. The strategy of ‘children eat first’ is particularly important for pregnant women because suboptimal antenatal nutrition is linked to an increased risk of adverse pregnancy outcomes such as low birth weight and preterm birth [91]. Of crucial and unique importance here is that maternal coping strategies adopted to protect children born, when used during pregnancy, may have a dual effect in that they potentially adversely impact the unborn child, with the potential for intergenerational impact across the life course period. Understanding the way women prioritise their children's food and nutrition needs is crucial in designing effective interventions aimed at managing food insecurity in pregnancy. Successful strategies targeting food insecurity in pregnancy must consider family and household needs and be specific to individual family units and preferences.

### 4.1 Strengths and Limitations

This review is the first to summarise the qualitative experiences of pregnant women who are food insecure within HICs since the global financial crisis of 2008. The review had consistent use of rigorous methodologies in the development of the protocol and conducting and reporting of the review by following the ENTREQ checklist [25] as well as using the GRADE CERQual approach [40] to assess confidence in our themes. A strength of the review's findings is that women reported similar experiences across studies settings/countries in varying economic, social and cultural contexts. Whilst the qualitative synthesis was limited to an extent by the original authors' pre‐selection of quotations, the thematic analysis used raw quotations throughout with the aim of creating an original synthesis. However, authors would occasionally highlight a key insight of pregnant women's experiences but would not include sufficient quotations that had led them to this interpretation. Meanwhile, the qualitative data we were able to extract preclude any direct pregnancy versus pre‐pregnancy comparison. This was particularly true for studies where food insecurity was implied and, therefore, not a key focus of the paper. Any potential bias from the authors' own interpretation, with no raw evidence, was addressed with less weighting given to those studies during synthesis. A further limitation of the review was the lack of identified data from across all HICs. All studies were set in Western HIC contexts. Inclusion of Western HIC only was not intentional; we identified no studies from other HICs that met our inclusion criteria. Moreover, 10 studies were based in Europe (seven in the United Kingdom, one in France, one in Denmark and one in Germany), with two studies originating in Australia. In comparison, 20 studies were set in North America, primarily the USA (*n* = 15). The review is, therefore, not equally weighted in a global context of HICs and more primary research is needed with pregnant women across a wider range of HICs. Finally, whilst we did not identify any non‐English language studies that met the inclusion criteria for this review (perhaps in itself an artefact of the weighting of published qualitative literature on this topic to Western HIC contexts), it is important to reflect upon our decision to use Google Translate during title and abstract screening to mitigate against potential language bias. Whilst use of automated translation software, including Google Translate, is a widely accepted process during screening phases of published systematic reviews, doing so is not without limitation. For example, Aiken has highlighted that Google Translate is more accurate at translating some languages over others [92], whilst Balk et al. [93] suggest viewing use as a potential trade‐off between completeness (not overlooking eligible studies) and risk of error (due to inaccurate translation).

## 5 Conclusions

Food insecurity in pregnancy is a significant health issue impacting women and children. This meta‐synthesis of qualitative studies focused on the experience of food insecurity during pregnancy across HICs, a life course period with unique structural factors increasing the risk of food insecurity and increased vulnerabilities to the adverse outcomes associated with food insecurity due to increased nutritional demands. It illustrates that food insecurity may impair the ability of pregnant women to consume a diet that meets their nutritional needs. This research, therefore, contributes evidence to support the need to screen and monitor the prevalence of food insecurity in pregnancy. Review findings also emphasise how important it is for clinicians, in a variety of settings across HICs, to assess the food security status of their patients as a routine aspect of care in order to support women to mitigate the impacts of food insecurity and its underlying causes to improve postpartum health and wellbeing.

## Author Contributions

Nicola Heslehurst, Steph Scott, Giang Nguyen and Lucy Clark designed the research. All authors contributed to data collection, including data searches, screening, data extraction and quality appraisal. Steph Scott, Zoë Bell and Lucy Clark analysed the data. All authors contributed to writing sections of the manuscript, and all authors reviewed and commented on the subsequent and final drafts.

## Funding

The authors have nothing to report.

## Conflicts of Interest

The authors declare no conflicts of interest.

## Supporting information

Supporting File

## Data Availability

The data that support the findings of this study are available from the corresponding author upon reasonable request.

## References

[1] S. A. Anderson , “Core Indicators of Nutritional State for Difficult‐to‐Sample Populations,” Journal of Nutrition 120 (1990): 1555–1598. 10.1093/jn/120.suppl_11.1555 2243305

[2] J. Clapp , W. G. Moseley , B. Burlingame , and P. Termine , “Viewpoint: The Case for a Six‐Dimensional Food Security Framework,” Food Policy 106 (2022): 102164.

[3] J. Blackshaw , M. Ewins , and M. Chang , “Health Matters: Addressing the Food Environment as Part of a Local Whole Systems Approach to Obesity,” UK Health Security Agency Blog, 8 August, 2019, https://ukhsa.blog.gov.uk/2019/08/08/health-matters-addressing-the-food-environment-as-part-of-a-local-whole-systems-approach-to-obesity/.

[4] C. Pineau , P. L. Williams , J. Brady , M. Waddington , and L. Frank , “Exploring Experiences of Food Insecurity, Stigma, Social Exclusion, and Shame Among Women in High‐Income Countries: A Narrative Review,” Canadian Food Studies La Revue canadienne des études sur l'alimentation 8, no. 3 (2021), 10.15353/cfs-rcea.v8i3.473.

[5] A. Drewnowski , “Food Insecurity Has Economic Root Causes,” Nature Food 3, no. 8 (2022): 555–556. 10.1038/s43016-022-00577-w PMC9362113 35965676

[6] The Food and Agriculture Organization , “The State of Food Security and Nutrition in the World 2020: Transforming Food Systems for Affordable Healthy Diets,” The State of Food Security and Nutrition in the World 2020 | UNICEF.

[7] C. M. Pollard and S. Booth , “Food Insecurity and Hunger in Rich Countries—It Is Time for Action Against Inequality,” International Journal of Environmental Research and Public Health 16, no. 10 (2019): 1804. 31117216 10.3390/ijerph16101804 PMC6572174

[8] J. Meadows , M. Montano , A. J. K. Alfar , et al., “The Impact of the Cost‐of‐Living Crisis on Population Health in the UK: Rapid Evidence Review,” BMC Public Health 24, no. 1 (2024): 561, 10.1186/s12889-024-17940-0. 38388342 PMC10882727

[9] R. M. Sumsion , H. M. June , and M. R. Cope , “The Impact of COVID‐19 on Food Security: A Review,” SN Social Sciences 3, no. 10 (2023): 176, 10.1007/s43545-023-00762-z.

[10] A. Ruckert and R. Labonté , “The Global Financial Crisis and Health Equity: Early Experiences From Canada,” Globalization and Health 10, no. 1 (2014): 2, 10.1186/1744-8603-10-2. 24393250 PMC3974147

[11] M. Vilar‐Compte , S. Sandoval‐Olascoaga , A. Bernal‐Stuart , S. Shimoga , and A. Vargas‐Bustamante , “The Impact of the 2008 Financial Crisis on Food Security and Food Expenditures in Mexico: A Disproportionate Effect on the Vulnerable,” Public Health Nutrition 18, no. 16 (2015): 2934–2942. 25428800 10.1017/S1368980014002493 PMC4534333

[12] G. C. Di Renzo and V. Tosto , Food Insecurity, Food Deserts, Reproduction and Pregnancy: We Should Alert From Now (Taylor & Francis, 2022), 9119–9121. 10.1080/14767058.2021.2016052 34918992

[13] G. Nguyen , Z. Bell , G. Andreae , et al., “Food Insecurity During Pregnancy in High‐Income Countries, and Maternal Weight and Diet: A Systematic Review and Meta‐Analysis,” Obesity Reviews 25, no. 7 (2024): e13753, 10.1111/obr.13753. 38693587

[14] Z. Bell , G. Nguyen , G. Andreae , et al., “Associations Between Food Insecurity in High‐Income Countries and Pregnancy Outcomes: A Systematic Review and Meta‐Analysis,” PLoS Medicine 21, no. 9 (2024): e1004450, 10.1371/journal.pmed.1004450. 39255262 PMC11386426

[15] K. L. Lindsay , C. Heneghan , B. McNulty , L. Brennan , and F. M. McAuliffe , “Lifestyle and Dietary Habits of an Obese Pregnant Cohort,” Maternal and Child Health Journal 19 (2015): 25–32. 24740724 10.1007/s10995-014-1491-2

[16] Z. Bell , S. Scott , S. Visram , J. Rankin , C. Bambra , and N. Heslehurst , “Experiences and Perceptions of Nutritional Health and Wellbeing Amongst Food Insecure Women in Europe: A Qualitative Meta‐Ethnography,” Social Science & Medicine (1982) 311 (2022): 115313. 36087388 10.1016/j.socscimed.2022.115313

[17] A. Bastian , C. Parks , A. Yaroch , et al., “Factors Associated With Food Insecurity Among Pregnant Women and Caregivers of Children Aged 0–6 Years: A Scoping Review,” Nutrients 14, no. 12 (2022): 2407. 35745136 10.3390/nu14122407 PMC9227310

[18] E. Mane , A. M. Giaquinto , C. Cafiero , S. Viviani , and G. Anriquez , “Why Are Women More Food Insecure Than Men?,” in Exploring Socio‐Economic Drivers and the Role of COVID‐19 in Widening the Global Gender Gap (FAO, 2023), 10.4060/cc9160en.

[19] A. Nazmi , S. Martinez , A. Byrd , et al., “A Systematic Review of Food Insecurity Among US Students in Higher Education,” Journal of Hunger & Environmental Nutrition 14, no. 5 (2019): 725–740.

[20] R. Mansour , P. Liamputtong , and A. Arora , “Prevalence, Determinants, and Effects of Food Insecurity Among Middle Eastern and North African Migrants and Refugees in High‐Income Countries: A Systematic Review,” International Journal of Environmental Research and Public Health 17, no. 19 (2020): 7262. 33020437 10.3390/ijerph17197262 PMC7579266

[21] C. Easton , A. Oudshoorn , T. Smith‐Carrier , C. Forchuk , and C. A. Marshall , “The Experience of Food Insecurity During and Following Homelessness in High‐Income Countries: A Systematic Review and Meta‐Aggregation,” Health & Social Care in the Community 30, no. 6 (2022): 3384. 10.1111/hsc.13939 35869792

[22] K. Sinclair , D. Ahmadigheidari , D. Dallmann , M. Miller , and H. Melgar‐Quiñonez , “Rural Women: Most Likely to Experience Food Insecurity and Poor Health in Low‐and Middle‐Income Countries,” Global Food Security 23 (2019): 104–115.

[23] M. Maynard , L. Andrade , S. Packull‐McCormick , C. M. Perlman , C. Leos‐Toro , and S. I. Kirkpatrick , “Food Insecurity and Mental Health Among Females in High‐Income Countries,” International Journal of Environmental Research and Public Health 15, no. 7 (2018): 1424. 29986420 10.3390/ijerph15071424 PMC6068629

[24] S. Iqbal and I. Ali , “Maternal Food Insecurity in Low‐Income Countries: Revisiting Its Causes and Consequences for Maternal and Neonatal Health,” Journal of Agriculture and Food Research 3 (2021): 100091.

[25] A. Tong , K. Flemming , E. McInnes , S. Oliver , and J. Craig , “Enhancing Transparency in Reporting the Synthesis of Qualitative Research: ENTREQ,” BMC Medical Research Methodology 12, no. 1 (2012): 181, 10.1186/1471-2288-12-181. 23185978 PMC3552766

[26] L. Clark , S. Scott , G. Nguyen , et al., “Women's Experiences of Food Insecurity During Pregnancy in High Income Countries: A Qualitative Systematic Review,” PROSPERO (2024), https://www.crd.york.ac.uk/PROSPERO/view/CRD42023404774.

[27] https://foodfoundation.org.uk/.

[28] The Trussell Trust, accessed 4 November 2024, https://www.trussell.org.uk/we-need-your-support-ppc?/&gad_source=1&gclid=CjwKCAiAudG5BhAREiwAWMlSjGWOj6PEUXRMHZjh982Zu17bmAXR6HYOL6sFOjBWYeSAZknf5hgLfxoCQDcQAvD_BwE&gclsrc=aw.ds.

[29] The King's Fund, accessed 4 November 2024, https://www.kingsfund.org.uk/.

[30] The World Health Organisation, accessed 4 November 2024, https://www.who.int/data/#highlights.

[31] N. R. Haddaway , M. J. Grainger , and C. T. Gray , “Citationchaser: A Tool for Transparent and Efficient Forward and Backward Citation Chasing in Systematic Searching,” Research Synthesis Methods 13, no. 4 (2022): 533–545. 35472127 10.1002/jrsm.1563

[32] EndNote21, “The EndNote Team: Clarivate.”

[33] M. Ouzzani , H. Hammady , Z. Fedorowicz , and A. Elmagarmid , “Rayyan—A Web and Mobile App for Systematic Reviews,” Systematic Reviews 5 (2016): 210. 27919275 10.1186/s13643-016-0384-4 PMC5139140

[34] M. J. Page , J. E. McKenzie , P. M. Bossuyt , et al., “The PRISMA 2020 Statement: An Updated Guideline for Reporting Systematic Reviews,” BMJ 372 (2021): n71, 10.1136/bmj.n71. 33782057 PMC8005924

[35] M. Amir‐Behghadami , “SPIDER as a Framework to Formulate Eligibility Criteria in Qualitative Systematic Reviews,” BMJ Supportive & Palliative Care 14, no. e1 (2024): e312–e313. 10.1136/bmjspcare-2021-003161 33975828

[36] The World Bank , “High‐Income Economies,” accessed November 4, 2024, https://data.worldbank.org/country/XD.

[37] Programme CAS, “CASP Checklist: 10 Questions to Help Make Sense of Qualitative Research 2018.”

[38] J. Thomas and A. Harden , “Methods for the Thematic Synthesis of Qualitative Research in Systematic Reviews,” BMC Medical Research Methodology 8 (2008): 45. 18616818 10.1186/1471-2288-8-45 PMC2478656

[39] E. Nicholson , T. Murphy , P. Larkin , C. Normand , and S. Guerin , “Protocol for a Thematic Synthesis to Identify Key Themes and Messages From a Palliative Care Research Network,” BMC Research Notes 9 (2016): 478. 27769317 10.1186/s13104-016-2282-1 PMC5073737

[40] S. Lewin , A. Booth , C. Glenton , et al., “Applying GRADE‐CERQual to Qualitative Evidence Synthesis Findings: Introduction to the Series,” supplement, Implementation Science 13, no. Suppl 1 (2018): 2. 29384079 10.1186/s13012-017-0688-3 PMC5791040

[41] S. Allen , A. Dev , C. Canavan , and D. Goodman , “Intersecting Substance Use Disorder and Unmet Social Needs in Rural Pregnant Women,” Journal of Obstetric, Gynecologic, and Neonatal Nursing: JOGNN 53, no. 5 (2024): 485–490. 38796173 10.1016/j.jogn.2024.04.006

[42] S. Allen , W. M. Onsando , I. Patel , C. Canavan , D. Goodman , and A. Dev , “Food Insecurity and Food Access Among Women in Northern New England During the Perinatal Period,” Journal of Obstetric, Gynecologic, and Neonatal Nursing: JOGNN 52, no. 5 (2023): 374–383. 37524310 10.1016/j.jogn.2023.06.004

[43] S. Booth , C. Deen , K. Thompson , et al., “Conceptualisation, Experiences and Suggestions for Improvement of Food Security Amongst Aboriginal and Torres Strait Islander Parents and Carers in Remote Australian Communities,” Social Science & Medicine (1982) 320 (2023): 115726. 36753996 10.1016/j.socscimed.2023.115726

[44] S. Cumming and A. Symon , “Pregnant and Homeless in the UK: A Qualitative Analysis of Maternal Experiences in Temporary Accommodation,” Birth 52, no. 3 (2025): 503–510. 40183469 10.1111/birt.12919 PMC12434225

[45] M. Graham , K. Uesugi , and C. Olson , “Barriers to Weight‐Related Health Behaviours: A Qualitative Comparison of the Socioecological Conditions Between Pregnant and Post‐Partum Low‐Income Women,” Maternal & Child Nutrition 12, no. 2 (2016): 349–361, 10.1111/mcn.12135. 25040706 PMC4556594

[46] R. S. Gross , A. L. Mendelsohn , M. M. Arana , and M. J. Messito , “Food Insecurity During Pregnancy and Breastfeeding by Low‐Income Hispanic Mothers,” Pediatrics 143, no. 6 (2019): e20184113, 10.1542/peds.2018-4113. 31088893 PMC6564052

[47] J. Marshall , L. Davies , L. Hdrc , et al., “A Qualitative Exploration of Women's Experiences of Food Insecurity Around Pregnancy Aligned With the Socio‐Ecological Model,” Appetite 216 (2026): 108264. 40848942 10.1016/j.appet.2025.108264

[48] S. Oresnik , T. Moffat , L. McKerracher , and D. M. Sloboda , “A Syndemic Perspective on Food Insecurity, Gestational Diabetes, and Mental Health Disorders During Pregnancy,” Social Science & Medicine (1982) 373 (2025): 117994. 40158449 10.1016/j.socscimed.2025.117994

[49] M. Quintanilha , M. J. Mayan , M. Jarman , and R. C. Bell , “Prevalence and Experiences of Food Insecurity Among Immigrant Women Connected to Perinatal Programs at a Community‐Based Organization in Edmonton, Canada,” International Journal of Migration, Health and Social Care 15, no. 2 (2019): 121–132, 10.1108/ijmhsc-09-2018-0064.

[50] S. M. Sim , S. F. L. Kirk , and M. Aston , “Mothering at the Intersection of Marginality: Exploring Breastfeeding Beliefs and Practices Among Women From Nova Scotia, Canada Who Identify as Overweight, Low Income, and Food Insecure,” Qualitative Health Research 30, no. 11 (2020): 1737–1748, 10.1177/1049732320921830. 32452301

[51] H. Waberi , C. Ehlert , G. Ibrahim , H. Egal , and L. McKerracher , “Food (in) Security and Peripartum Health in Marginalised Neighbourhoods in Denmark: Intersectional and Biopsychosocial Perspectives From Birthing Parents and Care Workers,” Sociology of Health & Illness 47, no. 7 (2025): e70070. 40768340 10.1111/1467-9566.70070 PMC12327573

[52] L. M. Yee , J. M. McGuire , S. M. Taylor , C. M. Niznik , and M. A. Simon , “Social and Environmental Barriers to Nutrition Therapy for Diabetes Management Among Underserved Pregnant Women: A Qualitative Analysis,” Journal of Nutrition Education and Behavior 48, no. 3 (2016): 170–180.e1, 10.1016/j.jneb.2015.11.003. 26706027

[53] J. Zinga , F. H. McKay , R. Lindberg , and P. van der Pligt , “Experiences of Food‐Insecure Pregnant Women and Factors Influencing Their Food Choices,” Maternal and Child Health Journal 26, no. 7 (2022): 1434–1441, 10.1007/s10995-022-03440-3. 35460501 PMC9034444

[54] C. K. Anderson , T. J. Walch , S. M. Lindberg , A. M. Smith , S. R. Lindheim , and L. D. Whigham , “Excess Gestational Weight Gain in Low‐Income Overweight and Obese Women: A Qualitative Study,” Journal of Nutrition Education and Behavior 47, no. 5 (2015): 404–411.e1, 10.1016/j.jneb.2015.05.011. 26187348 PMC4590982

[55] F. Arshad , M. Haith‐Cooper , and P. Palloti , “The Experiences of Pregnant Migrant Women in Detention: A Qualitative Study,” British Journal of Midwifery 26, no. 9 (2018): 591–596, 10.12968/bjom.2018.26.9.591.

[56] G. Borghi , P. Caillet , S. Devriendt , M. Lebeaupin , M. Poirier , and J.‐D. Poveda , “The Perceived Impact of Homelessness on Health During Pregnancy and the Postpartum Period: A Qualitative Study Carried out in the Metropolitan Area of Nantes, France,” PLoS One 18, no. 2 (2023): e0280273. 36724156 10.1371/journal.pone.0280273 PMC9891509

[57] T. C. J. Burton , N. Crooks , L. Pezley , et al., “Food Choice and Dietary Perspectives of Young, Urban, Black Pregnant Women: A Focus Group Study,” Nutrients 16, no. 6 (2024): 781. 38542692 10.3390/nu16060781 PMC10974382

[58] R. Dundas , M. Boroujerdi , S. Browne , et al., “Evaluation of the Healthy Start Voucher Scheme on Maternal Vitamin Use and Child Breastfeeding: A Natural Experiment Using Data Linkage,” Public Health Res 11, no. 11 (2023), 10.3310/RTEU2107. 37953640

[59] R. Ellul , R. McCarthy , and M. Haith‐Cooper , “Destitution in Pregnancy: Forced Migrant Women's Lived Experiences,” British Journal of Midwifery 28, no. 11 (2020): 778–787, 10.12968/bjom.2020.28.11.778.

[60] S. Evenosky , E. Lewis , and K. I. DiSantis , “A Mixed Methods Case Study of Food Shopping in a Community With High Infant Mortality,” Nutrients 13, no. 11 (2021): 3845, 10.3390/nu13113845. 34836108 PMC8623881

[61] S. C. Gewalt , S. Berger , J. Szecsenyi , and K. Bozorgmehr , “‘If You Can, Change This System’ ‐Pregnant Asylum Seekers' Perceptions on Social Determinants and Material Circumstances Affecting Their Health Whilst Living in State‐Provided Accommodation in Germany ‐ A Prospective, Qualitative Case Study,” BMC Public Health 19, no. 1 (2019): 287, 10.1186/s12889-019-6481-2. 30866874 PMC6417255

[62] S. W. Groth , A. H. Simpson , and I. D. Fernandez , “The Dietary Choices of Women Who Are Low‐Income, Pregnant, and African American,” Journal of Midwifery & Women's Health 61, no. 5 (2016): 606–612. 10.1111/jmwh.12463 27448099

[63] A. Hromi‐Fiedler , D. Chapman , S. Segura‐Pérez , et al., “Barriers and Facilitators to Improve Fruit and Vegetable Intake Among WIC‐Eligible Pregnant Latinas: An Application of the Health Action Process Approach Framework,” Journal of Nutrition Education and Behavior 48, no. 7 (2016): 468–477.e1, 10.1016/j.jneb.2016.04.398. 27373861 PMC4934128

[64] H. Iqbal , J. West , R. R. C. McEachan , and M. Haith‐Cooper , “Identifying the Health Concerns of Pregnant British Pakistani Women Living in Deprived Areas: A Qualitative Study,” Maternal and Child Health Journal 28, no. 3 (2024): 489–495. 37902920 10.1007/s10995-023-03797-z PMC10914889

[65] L. McKerracher , S. Oresnik , T. Moffat , et al., “Addressing Embodied Inequities in Health: How Do We Enable Improvement in Women's Diet in Pregnancy?,” Public Health Nutrition 23, no. 16 (2020): 2994–3004, 10.1017/s1368980020001093. 32627725 PMC10200536

[66] E. M. Nagourney , D. Goodman , Y. Lam , K. M. Hurley , J. Henderson , and P. J. Surkan , “Obese Women's Perceptions of Weight Gain During Pregnancy: A Theory‐Based Analysis,” Public Health Nutrition 22, no. 12 (2019): 2228–2236, 10.1017/s1368980019000703. 31134872 PMC10260617

[67] H. Ohly , N. Crossland , F. Dykes , N. M. Lowe , and V. H. Moran , “A Realist Qualitative Study to Explore How Low‐Income Pregnant Women Use Healthy Start Food Vouchers,” Maternal & Child Nutrition 15, no. 1 (2018): e12632, 10.1111/mcn.12632. 29956890 PMC7199019

[68] K. H. Paul , M. L. Graham , and C. M. Olson , “The Web of Risk Factors for Excessive Gestational Weight Gain in Low Income Women,” Maternal and Child Health Journal 17, no. 2 (2012): 344–351, 10.1007/s10995-012-0979-x. PMC4535446 22415812

[69] N. R. Reyes , A. A. Klotz , and S. J. Herring , “A Qualitative Study of Motivators and Barriers to Healthy Eating in Pregnancy for Low‐Income, Overweight, African‐American Mothers,” Journal of the Academy of Nutrition and Dietetics 113, no. 9 (2013): 1175–1181, 10.1016/j.jand.2013.05.014. 23871106 PMC3782301

[70] A. Struthers , C. Metge , C. Charette , et al., “Understanding the Particularities of an Unconditional Prenatal Cash Benefit for Low‐Income Women: A Case Study Approach,” Inquiry: A Journal of Medical Care Organization, Provision and Financing 56, no. NA (2019): 46958019870967, 10.1177/0046958019870967. 31434525 PMC6709438

[71] N. J. Wise , “Pregnant Adolescents, Beliefs About Healthy Eating, Factors That Influence Food Choices, and Nutrition Education Preferences,” Journal of Midwifery & Women's Health 60, no. 4 (2015): 410–418, 10.1111/jmwh.12275. 26255801

[72] C. M. Whisner , M. Bruening , and K. O. O'Brien , “A Brief Survey of Dietary Beliefs and Behaviors of Pregnant Adolescents,” Journal of Pediatric and Adolescent Gynecology 29, no. 5 (2016): 476–481, 10.1016/j.jpag.2016.03.002. 26995508 PMC10676290

[73] H. Burchett and A. Seeley , “Short Report: Good Enough to Eat? The Diet of Pregnant Teenagers,” International Journal of Health Promotion and Education 41, no. 2 (2003): 59–61.

[74] D. Ravikumar , E. Spyreli , J. Woodside , M. McKinley , and C. Kelly , “Parental Perceptions of the Food Environment and Their Influence on Food Decisions Among Low‐Income Families: A Rapid Review of Qualitative Evidence,” BMC Public Health 22, no. 1 (2022): 9, 10.1186/s12889-021-12414-z. 34983469 PMC8727174

[75] A. Drewnowski , “Obesity, Diets, and Social Inequalities,” Nutrition Reviews 67, no. Suppl 1 (2009): S36–S39, 10.1111/j.1753-4887.2009.00157.x. 19453676

[76] H. J. McKenzie and F. H. McKay , “Food as a Discretionary Item: The Impact of Welfare Payment Changes on Low‐Income Single Mother's Food Choices and Strategies,” Journal of Poverty and Social Justice 25, no. 1 (2017): 35–48, 10.1332/175982716x14822521840954.

[77] P. F. van der Pligt , K. Kuswara , S. A. McNaughton , et al., “Maternal Diet Quality and Associations With Plasma Lipid Profiles and Pregnancy‐Related Cardiometabolic Health,” European Journal of Nutrition 62, no. 8 (2023): 3369–3381, 10.1007/s00394-023-03244-3. 37646831 PMC10611854

[78] M. Li , J. Grewal , S. N. Hinkle , et al., “Healthy Dietary Patterns and Common Pregnancy Complications: A Prospective and Longitudinal Study,” American Journal of Clinical Nutrition 114, no. 3 (2021): 1229–1237, 10.1093/ajcn/nqab145. 34075392 PMC8408886

[79] Y. Yu , I. Hardy , W. Sun , et al., “Association Between Diet Quality During Preconception or Pregnancy and Adverse Perinatal Outcomes: A Systematic Review and Meta‐Analysis,” Authorea, ahead of print, September 24, 2021, 10.22541/au.163251147.74307797/v1.

[80] R. Pretorius and D. Palmer , “High‐Fiber Diet During Pregnancy Characterized by More Fruit and Vegetable Consumption,” Nutrients 13, no. 1 (2020): 35, 10.3390/nu13010035. 33374192 PMC7824257

[81] P. Fielding‐Singh and M. Cooper , “Negotiating Good Motherhood: Foodwork, Emotion Work, and Downscaling,” Journal of Marriage and Family 86, no. 1 (2024): 245–267, 10.1111/jomf.12934.

[82] L. Dean , B. Churchill , and L. Ruppanner , “The Mental Load: Building a Deeper Theoretical Understanding of How Cognitive and Emotional Labor Overload Women and Mothers,” Community, Work & Family 25, no. 1 (2022): 13–29, 10.1080/13668803.2021.2002813.

[83] M. S. Cardwell , “Stress: Pregnancy Considerations,” Obstetrical & Gynecological Survey 68, no. 2 (2013): 119–129, 10.1097/OGX.0b013e31827f2481. 23417218

[84] M. E. Coussons‐Read , “Effects of Prenatal Stress on Pregnancy and Human Development: Mechanisms and Pathways,” Obstetric Medicine 6, no. 2 (2013): 52–57, 10.1177/1753495x12473751. 27757157 PMC5052760

[85] C. Dunkel Schetter and L. Tanner , “Anxiety, Depression and Stress in Pregnancy: Implications for Mothers, Children, Research, and Practice,” Current Opinion in Psychiatry 25, no. 2 (2012): 141–148, 10.1097/YCO.0b013e3283503680. 22262028 PMC4447112

[86] M. J. Meaney , M. Szyf , and J. R. Seckl , “Epigenetic Mechanisms of Perinatal Programming of Hypothalamic‐Pituitary‐Adrenal Function and Health,” Trends in Molecular Medicine 13, no. 7 (2007): 269–277, 10.1016/j.molmed.2007.05.003. 17544850

[87] K. L. Radimer , C. M. Olson , J. C. Greene , C. C. Campbell , and J.‐P. Habicht , “Understanding Hunger and Developing Indicators to Assess It in Women and Children,” Journal of Nutrition Education 24, no. 1 (1992): 36S–44S.

[88] M. A. Martin and A. M. Lippert , “Feeding Her Children, but Risking Her Health: The Intersection of Gender, Household Food Insecurity and Obesity,” Social Science & Medicine (1982) 74, no. 11 (2012): 1754–1764, 10.1016/j.socscimed.2011.11.013. 22245381 PMC3338899

[89] Y. Luo , C. Mobley , L. Hossfeld , et al., “The Association Between Food Insecurity and Making Hunger‐Coping Trade‐Offs During the COVID‐19 Pandemic: The Role of Sources of Food and Easiness in Food Access,” Nutrients 14, no. 21 (2022): 4616. 36364877 10.3390/nu14214616 PMC9658505

[90] A. A. Zekeri , “Livelihood Strategies of Food‐Insecure Poor, Female‐Headed Families in Rural Alabama,” Psychological Reports 101, no. 3 pt. 2 (2007): 1031–1036, 10.2466/pr0.101.4.1031-1036. 18361115

[91] C. Mearig , “Complications & Symptoms of Not Eating Enough During Pregnancy: First Trimester & On,” 2023, accessed November 10, 2024, https://zayacare.com/blog/not-eating-enough-during-pregnancy/#:~:text=Inadequate%20nutrition%20can%20increase%20the,health%20problems%20for%20the%20baby.

[92] M. Aiken , “An Updated Evaluation of Google Translate Accuracy,” Studies in Linguistics and Literature 3, no. 3 (2019): p253.

[93] E. M. Balk , M. Chung , M. L. Chen , L. K. W. Chang , and T. A. Trikalinos , “Data Extraction From Machine‐Translated Versus Original Language Randomized Trial Reports: A Comparative Study,” Systematic Reviews 2, no. 1 (2013): 97. 24199894 10.1186/2046-4053-2-97 PMC4226266

