# Supplementary material for: Experiences of Food Insecurity During Pregnancy in High‐Income Countries: A Meta‐Synthesis of Qualitative Studies
Source: J Hum Nutr Diet. 2026 Apr 13;39(2):e70244. doi: 10.1111/jhn.70244 (PMC13076677; doi:10.1111/jhn.70244)
Supplement: Supplementary file 1 — Supporting File [file JHN-39-0-s001.docx]

**Online Supplementary Tables**

**Supplementary tables:**

Table S1. Translation of search terms across databases for a meta-synthesis of qualitative studies exploring pregnant women’s experiences of food insecurity in high-income countries (HICs)

Table S2. Grey literature website searches for a meta-synthesis of qualitative studies exploring pregnant women’s experiences of food insecurity in high-income countries (HICs)

Table S3. Critical Appraisal Skills Program (CASP) Qualitative Checklist

Table S4. Additional study characteristics extracted from included studies exploring pregnant women’s experiences of food insecurity in high-income countries (HICs) (n=32)

Table S5: Description of themes and sub-themes across included studies exploring pregnant women’s experiences of food insecurity in high-income countries (HICs) and CERQual assessment of confidence (n=32)

**Table S1: Translation of search terms across databases for a meta-synthesis of qualitative studies exploring pregnant women’s experiences of food insecurity in high-income countries (HICs)**

| **Database** | **Search terms** |
| --- | --- |
| Ovid MEDLINE(R) 1946 to 16 January 2026 | 1. exp pregnancy/  2. pregnan*.mp.  3. expect* mother*.mp.  4. gestation.mp.  5. exp pregnancy trimesters/  6. childbear*.mp.  7. child bear*.mp.  8. maternal.mp.  9. prenatal.mp.  10. perinatal.mp.  11. antenatal.mp.  12. ante natal.mp.  13. exp perinatal care/  14. peripartum.mp.  15. antepartum.mp.  16. ante partum.mp.  17. 1 or 2 or 3 or 4 or 5 or 6 or 7 or 8 or 9 or 10 or 11 or 12 or 13 or 14 or 15 or 16  18. Qualitative.mp.  19. exp qualitative research/  20. exp interview/  21. interview*.mp.  22. personal experience.mp.  23. narrative.mp.  24. focus group.mp.  25. exp focus groups/  26. case stud*.mp.  27. thematic analysis.mp.  28. thematic synthesis.mp.  29. phenomenologic*.mp.  30. phenomenology.mp.  31. ethnograph*.mp.  32. grounded theory.mp.  33. 18 or 19 or 20 or 21 or 22 or 23 or 24 or 25 or 26 or 27 or 28 or 29 or 30 or 31 or 32  34. exp food insecurity/  35. food insecurit*.mp.  36. (food adj4 insecurit*).mp.  37. food poverty.mp.  38. food deprivation.mp.  39. (food adj3 depriv*).mp.  40. foodbank*.mp.  41. food bank.mp.  42. food assistance.mp.  43. food stamps.mp.  44. food choice.mp.  45. food insufficient.mp.  46. food access.mp.  47. food provision.mp.  48. food aid.mp.  49. food shortage.mp.  50. food stress.mp.  51. food anxiety.mp.  52. food uncertainty.mp.  53. food desert*.mp.  54. exp hunger/  55. hung*.mp.  56. starv*.mp.  57. nutri* deficien*.mp.  58. 34 or 35 or 36 or 37 or 38 or 39 or 40 or 41 or 42 or 43 or 44 or 45 or 46 or 47 or 48 or 49 or 50 or 51 or 52 or 53 or 54 or 55 or 56 or 57  59. exp diet/  60. diet*.mp.  61. exp malnutrition/  62. malnutrition.mp.  63. nutri*.mp.  64. exp food/  65. food*.mp.  66. eat.mp.  67. snack*.mp.  68. healthy eating.mp.  69. balanced eating.mp.  70. exp meals/  71. meal*.mp.  72. exp obesity/  73. obesity.mp.  74. nourish*.mp.  75. (food adj3 intake).mp.  76. undernourish*.mp.  77. overnourish*.mp.  78. overweight.mp.  79. 59 or 60 or 61 or 62 or 63 or 64 or 65 or 66 or 67 or 68 or 69 or 70 or 71 or 72 or 73 or 74 or 75 or 76 or 77 or 78  80. 58 or 79  81. 17 and 33 and 80  82. limit 81 to yr="2008 -Current”  Qualitative filter applied: limit to “qualitative (best balance of sensitivity and specificity). |
| Ovid Embase 1974 to 16 January 2026 | 1. Exp Pregnancy/  2. Pregnan*.mp.  3. Expect* mother*.mp.  4. Gestation.mp.  5. Pregnan* trimester*.mp.  6. Childbear*.mp.  7. Child bear*.mp.  8. Exp maternal care/  9. Maternal.mp.  10. Prenatal.mp.  11. Exp prenatal care/  12. Perinatal.mp.  13. Exp perinatal care/  14. Antenatal.mp  15. Ante natal.mp.  16. Peripartum.mp.  17. Antepartum.mp.  18. Ante partum.mp.  19. 1 or 2 or 3 or 4 or 5 or 6 or 7 or 8 or 9 or 10 or 11 or 12 or 13 or 14 or 15 or 16 or 17 or 18  20. Qualitative.mp.  21. Exp qualitative research/  22. exp interview/  23. Interview*.mp.  24. Narrative.mp.  25. Focus group.mp.  26. Case stud*.mp.  27. Thematic analysis.mp.  28. Thematic synthesis.mp.  29. Phenomenologic*.mp.  30. Exp phenomenology/  31. Ethnograph*.mp.  32. Grounded theory.mp.  33. 20 or 21 or 22 or 23 or 24 or 25 or 26 or 27 or 28 or 29 or 30 or 31 or 32  34. Exp food insecurity/  35. Food insecurit*.mp.  36. (food adj4 insecurit*).mp.  37. Food poverty.mp.  38. Exp food deprivation/  39. Food deprivation.mp.  40. (food adj3 depriv*).mp.  41. Foodbank*.mp.  42. Food assistance.mp.  43. Food stamps.mp.  44. Food choice.mp.  45. Food insufficient.mp.  46. Food access.mp.  47. Food provision.mp.  48. Food aid.mp.  49. Food shortage.mp.  50. Food stress.mp.  51. Food anxiety.mp.  52. Food uncertainty.mp.  53. Food desert.mp.  54. Hung*.mp.  55. Starv*.mp.  56. Exp nutritional deficiency/  57. Nutri* deficien*.mp.  58. 34 or 35 or 36 or 37 or 38 or 39 or 40 or 41 or 42 or 43 or 44 or 45 or 46 or 47 or 48 or 49 or 50 or 51 or 52 or 53 or 54 or 55 or 56 or 57  59. Exp nutrition/  60. Nutri*.mp.  61. Exp malnutrition/  62. Malnutrition.mp.  63. Exp food/  64. Food*.mp.  65. Exp diet/  66. Diet*.mp.  67. Eat.mp.  68. Snack*.mp.  69. Healthy eating.mp.  70. Balanced eating.mp.  71. Exp eating habit/  72. Portion size.mp.  73. Meal*.mp.  74. Exp obesity/  75. Obesity.mp.  76. nourish*.mp.  77. (food adj3 intake).mp.  78. undernourish*.mp.  79. overnourish*.mp.  80. overweight.mp.  81. 59 or 60 or 61 or 62 or 63 or 64 or 65 or 66 or 67 or 68 or 69 or 70 or 71 or 72 or 73 or 74 or 75 or 76 or 77 or 78 or 79 or 80  82. 81 or 58  83. 19 and 33 and 82  84. Limit 83 to yr=”2008-current”  Qualitative filter applied: “(best balance of sensitivity and specificity)”. |
| Elsevier Scopus 01 January 2008 to 16 January 2026 | Search Query:  ( ( TITLE-ABS-KEY ( pregnan* OR gestation* OR "expect* mother*" OR "pregnan* trimester*" OR childbear* OR "child bear*" OR maternal* OR prenatal* OR perinatal* OR antenatal* OR "ante natal*" OR peripartum* OR antepartum* OR "ante partum*" ) AND TITLE-ABS-KEY ( "qualitative research*" OR qualitative* OR interview* OR narrative* OR "focus group*" OR "case stud*" OR "thematic synthesis*" OR "thematic analysis*" OR phenomenolog* OR ethnograph* OR "grounded theory*" OR "personal experience*" ) ) ) AND ( ( TITLE-ABS-KEY ( "food insecuirt*" OR "food secur*" OR "food poverty*" OR "food depriv*" OR "food bank*" OR "food assistance" OR "food insufficienc*" OR "food access*" OR "food afford*" OR "food shortage*" OR "food desert*" OR "food aid*" OR "food provision*" OR "food anxiety*" OR "food stress*" OR "food uncertainty*" OR hung* OR foodbank* OR starv* OR "nutri* deficien*" ) ) OR ( TITLE-ABS-KEY ( nutrition* OR malnutrition* OR food* OR diet* OR eat* OR snack* OR "healthy eating*" OR "balanced eating*" OR "eating habit*" OR "portion size*" OR meal* OR obesity* OR overweight* OR undernourish* OR overnourish* OR nourish* ) ) ) AND PUBYEAR > 2007 AND PUBYEAR < 2024  Search 1 (pregnancy and qualitative)  TITLE-ABS-KEY ( pregnan* OR gestation* OR "expect* mother*" OR "pregnan* trimester*" OR childbear* OR "child bear*" OR maternal* OR prenatal* OR perinatal* OR antenatal* or "ante natal*" OR postpartum* OR antepartum* OR "ante partum*" )  AND TITLE-ABS-KEY ( "qualitative research*" OR qualitative* OR interview* OR narrative* OR "focus group*" OR "case stud*" OR "thematic synthesis*" OR "thematic analysis*" OR phenomenolog* OR ethnograph* OR "grounded theory*" OR “personal experience*” )  Search 2 (food insecurity)  TITLE-ABS-KEY ( "food insecuirt*" OR "food secur*" OR "food poverty*" OR "food depriv*" OR "food bank*" OR "food assistance" OR "food insufficienc*" OR "food access*" OR "food afford*" OR "food shortage*" OR "food desert*" OR "food aid*" OR "food provision*" OR "food anxiety*" OR "food stress*" OR “food choice*” OR "food uncertainty*" OR hung* OR foodbank* OR starv* OR "nutri* deficien*" )  Search 3 (diet)  TITLE-ABS-KEY (nutrition* OR malnutrition* OR food* OR diet* OR eat* OR snack* OR "healthy eating*" OR "balanced eating*" OR "eating habit*" OR "portion size*" OR meal* or obesity* OR overweight* OR nourish* OR overnourish* OR undernourish* )  Search 4 (food insecurity OR diet)  2 OR 3  Search 5 (pregnancy and qualitative and (food insecurity OR diet))  1 AND 4 |
| Clarivate Web of Science Core Collection 1970 to 16 January 2026 | Query #1  (TS=(pregnan* OR gestation* OR “expect* mother*” OR “pregnan* trimester*” OR childbear* OR “child bear*” OR maternal* OR prenatal* OR perinatal* OR antenatal* or “ante natal*” OR peripartum* OR antepartum* OR “ante partum*” )) AND TS=(“qualitative research*” OR qualitative* OR interview* OR narrative* OR “focus group*” OR “case stud*” OR “thematic synthesis*” OR “thematic analysis*” OR phenomenolog* OR ethnograph* OR “grounded theory*” OR “personal experience*” )  Query #2  TS=(“food insecuirt*” OR “food secur*” OR “food poverty*” OR “food depriv*” OR “food bank*” OR “food assistance” OR “food insufficienc*” OR “food access*” OR “food afford*” OR “food shortage*” OR “food desert*” OR “food aid*” OR “food provision*” OR “food anxiety*” OR “food stress*” OR “food uncertainty*” OR hung* OR foodbank* OR starv* OR “nutri* deficien*” OR “food stamps*” OR “food choices*” )  Query #3  (TS=(nutrition* OR malnutrition* OR food* OR diet* OR eat* OR snack* OR “healthy eating*” OR “balanced eating*” OR “eating habit*” OR “portion size*” OR meal* OR obesity* OR overweight* OR nourish* OR overnourish* OR undernourish* )))  Query #4  #2 OR #3  Search Query - #1 AND #4 |
| ProQuest ASSIA 1987 to 16 January 2026 | ((pregnan* OR gestation* OR “expect* mother*” OR “pregnan* trimester*” OR childbear* OR “child bear*” OR maternal* OR prenatal* OR perinatal* OR antenatal* or “ante natal*” OR peripartum* OR antepartum* OR “ante partum*” ) AND (“qualitative research*” OR qualitative* OR interview* OR narrative* OR “focus group*” OR “case stud*” OR “thematic synthesis*” OR “thematic analysis*” OR phenomenolog* OR ethnograph* OR “grounded theory*” OR “personal experience*” )) AND ((“food insecuirt*” OR “food secur*” OR “food poverty*” OR “food depriv*” OR “food bank*” OR “food assistance” OR “food insufficienc*” OR “food access*” OR “food afford*” OR “food shortage*” OR “food desert*” OR “food aid*” OR “food provision*” OR “food anxiety*” OR “food stress*” OR “food uncertainty*” OR hung* OR foodbank* OR starv* OR “nutri* deficien*” OR “food stamps*” OR “food choices*” ) OR (nutrition* OR malnutrition* OR food* OR diet* OR eat* OR snack* OR “healthy eating*” OR “balanced eating*” OR “eating habit*” OR “portion size*” OR meal* OR obesity* OR overweight* OR nourish* OR overnourish* OR undernourish* )) |
| EBSCO CINAHL 1981 to 16 January 2026 | S1  (MH “Pregnancy+”) OR (MH “Expectant Mothers”) OR (MH “Prenatal Care”) OR (MH “Perinatal Care”) OR (MH “Postnatal Care+”) OR (MH “Pregnancy Trimesters+”) OR “gestation* or childbear* or child bear* or maternal or prenatal or perinatal or ante natal* OR peripartum OR antepartum OR ante partum*”  AND (MH “Qualitative Studies+”) OR (MH “Interviews+”) OR (“Narratives+”) OR (MH “Focus Groups”) OR (“Case Studies”) OR (MH “Thematic Analysis”) OR (MH “Phenomenological Research”) OR (MH “Ethnographic Research”) OR (MH “Grounded Theory”) OR (MH “Life Experiences+”) OR “personal experience* or thematic synthesis or phenomenology or ethnography  S2  (MH “Food Security”) OR (MH “Food Preferences+”) OR (MH “Food Assistance”) OR (MH “Food Deserts”) OR (MH “Hunger”) OR (MH “Starvation”) OR “food insecurity or food poverty or food depriv* or food bank or foodbank or food insufficien* or food access or food afford or food shortage or food aid or food provision or food anxiety or food stress or food uncertainty or hung* or nutri* deficien* OR food stamp* OR food choice*”  S3  (MH “Nutrition+”) OR (MH “Malnutrition+”) OR (MH “Food+”) OR (MH “Diet+”) OR (MH “Obesity+”) OR (MH “Eating Behaviour”) OR (MH “Snacks”) OR (MH “Food Habits”) OR (MH “Portion Size”) OR (MH “Meals+”) OR “healthy eating OR balanced eating OR eating habit* OR obesity OR overweight OR nourish OR undernourish OR overnourish”  S4  S2 OR S3  S5  S1 AND S4 |

**Table S2: Grey literature website searches for a meta-synthesis of qualitative studies exploring pregnant women’s experiences of food insecurity in high-income countries (HICs)**

Search terms:

Pregnancy terms (‘pregnant’, ‘pregnancy’, ‘expecting mothers’) and food insecurity terms (‘food insecure’, ‘food’, ‘diet’, ‘food availability/access’).

| Name of website | URL | Date searched |
| --- | --- | --- |
| World Health Organization | <https://www.who.int/> | 03/02/2026 |
| The Trussell Trust (UK) | <https://www.trusselltrust.org/> | 03/02/2026 |
| The Food Foundation (UK) | <https://foodfoundation.org.uk/> | 03/02/2026 |
| The Kings Fund | <https://www.kingsfund.org.uk/> | 03/02/2026 |

**Table S3. Critical Appraisal Skills Program (CASP) Qualitative Checklist**

| **Section A Are the results valid?** | |
| --- | --- |
| 1. Was there a clear statement of the aims of the research? | Yes  No  Can’t Tell |
| *CONSIDER:*   - *what was the goal of the research?* - *why was it thought important?* - *its relevance* | |
| 1. Is a qualitative methodology appropriate? | Yes  No  Can’t Tell |
| *CONSIDER:*   - *If the research seeks to interpret or illuminate the actions and/or subjective experiences of research participants* - *Is qualitative research the right methodology for addressing the research goal?* | |
| 1. Was the research design appropriate to address the aims of the research? | Yes  No  Can’t Tell |
| *CONSIDER:*   - *if the researcher has justified the research design (e.g., have they discussed how they decided which method to use)* | |
| 1. Was the recruitment strategy appropriate to the aims of the research? | Yes  No  Can’t Tell |
| *CONSIDER:*   - *If the researcher has explained how the participants were selected* - *If they explained why the participants they selected were the most appropriate to provide access to the type of knowledge sought by the study* - *If there are any discussions around recruitment (e.g. why some people chose not to take part)* | |
| 1. Was the data collected in a way that addressed the research issue? | Yes  No  Can’t Tell |
| *CONSIDER:*   - *If the setting for the data collection was justified* - *If it is clear how data were collected (e.g. focus group, semi-structured interview etc.)* - *If the researcher has justified the methods chosen* - *If the researcher has made the methods explicit (e.g. for interview method, is there an indication of how interviews are conducted, or did they use a topic guide)* - *If methods were modified during the study. If so, has the researcher explained how and why* - *If the form of data is clear (e.g. tape recordings, video material, notes etc.)* - *If the researcher has discussed saturation of data* | |
| 1. Has the relationship between researcher and participants been adequately considered? | Yes  No  Can’t Tell |
| *CONSIDER:*   - *If the researcher critically examined their own role, potential bias and influence during (a) formulation of the research questions (b) data collection, including sample recruitment and choice of location* - *How the researcher responded to events during the study and whether they considered the implications of any changes in the research design* | |
| **Section B: What are the results?** | |
| 1. Have ethical issues been taken into consideration? | Yes  No  Can’t Tell |
| *CONSIDER:*   - *If there are sufficient details of how the research was explained to participants for the reader to assess whether ethical standards were maintained* - *If the researcher has discussed issues raised by the study (e.g. issues around informed consent or confidentiality or how they have handled the effects of the study on the participants during and after the study)* - *If approval has been sought from the ethics committee* | |
| 1. Was the data analysis sufficiently rigorous? | Yes  No  Can’t Tell |
| *CONSIDER:*   - *If there is an in-depth description of the analysis process* - *If thematic analysis is used. If so, is it clear how the categories/themes were derived from the data* - *Whether the researcher explains how the data presented were selected from the original sample to demonstrate the analysis process* - *If sufficient data are presented to support the findings* - *To what extent contradictory data are taken into account* - *Whether the researcher critically examined their own role, potential bias and influence during analysis and selection of data for presentation* | |
| 1. Is there a clear statement of findings? | Yes  No  Can’t Tell |
| *CONSIDER:*   - *If the findings are explicit* - *If there is adequate discussion of the evidence both for and against the researcher’s arguments* - *If the researcher has discussed the credibility of their findings (e.g. triangulation, respondent validation, more than one analyst)* - *If the findings are discussed in relation to the original research question* | |
| **Section C: Will the results help locally?** | |
| 1. How valuable is the research? | Yes  No  Can’t Tell |
| *CONSIDER:*   - *If the researcher discusses the contribution the study makes to existing knowledge or understanding (e.g., do they consider the findings in relation to current practice or policy, or relevant research-based literature* - *If they identify new areas where research is necessary* - *If the researchers have discussed whether or how the findings can be transferred to other populations or considered other ways the research may be used* | |
| **The papers are categorised as low, medium, or high based on their score out of 20. Each answer of Yes = 2, Can’t tell = 1, No = 0. Once all questions have been answered, if the paper has a score 20 it is high quality, 16-18 good quality and 15 or less is low quality.** | |

**Table S4. Additional study characteristics extracted from included studies exploring pregnant women’s experiences of food insecurity in high-income countries (HICs) (n=32)**

| Author, year, country | Method of data collection | Setting of recruitment | Inclusion/Exclusion criteria | Data analysis |
| --- | --- | --- | --- | --- |
| Allen et al, 2024^43^, USA | Semi-structured interviews conducted by telephone | Community health  resource specialist (CHRS) within hospital obstetrics and gynaecology department | Women who screened positively for food insecurity. Authors did not recontact participants for more information or interview additional participants – instead, original transcripts were reviewed for mention of substance use disorder or recovery. | Secondary data analysis using Thorne’s analytic expansion approach |
| Allen et al. 2023^44^, USA | Semi-structured interviews conducted by telephone | Community health  resource specialist (CHRS) within hospital obstetrics and gynaecology department | Women who screened positively for food insecurity | Reflexive Thematic analysis |
| Anderson et al. 2015^56^, USA | In-person Focus groups | Community-based perinatal centre in Madison | Pregnant or 6 weeks to 1-year postpartum, prenatal BMI >25, age >18 years, ability to speak English, and eligible for the Special Supplemental Nutrition Program for Women, Infants and Children (income falls below 185% of the US poverty income guidelines) | Mixed-methods approach to content analysis using both conventional and directed content analysis. |
| Arshad et al. 2018^57^, UK | In depth interviews using prompts to guide the direction of the conversation | Participants identified by cascading a request through voluntary sector networks; augmented by snowball sampling through friends and peers. | Over 18 yo, experienced some/all of pregnancy in detention, given birth in last 2 years, adequate English language or can bring own translator. Not been involved in a similar study in the last 2 years | Thematic analysis |
| Booth et al. 2023^45^, Australia | Semi-structured interviews | Part of a larger study focused on improving healthy food affordability and food security among Aboriginal and Torres Strait Islander women and children in remote Central Australia in the Northern Territory, and Cape York in Far North Queensland.  Community engagement led by two Aboriginal Community Controlled Health organisations who invited four communities (two control and two strategy) in each region to participate. | Inclusion in larger study, resident of Aboriginal and/or Torres Strait Islander, pregnant women or women breastfeeding children up to five years, and parents/carers of children aged six months to five years, who identified as a resident and planned to reside in the community for the next eight months. | Four stage thematic analysis |
| Borghi et al. 2023^58^, France | In-person, semi-structured interviews | Directly in the field through sites of homeless people including healthcare facilities, homeless hotels, overnight shelters and non-conventional dwellings in the community | Homeless pregnant or in the postpartum period (6 weeks following childbirth) at the time of recruitment or being a member of the social surroundings (family members and friends) of a homeless pregnant or postpartum woman, ii) over 18 yo or being a minor with an attested consent to participate in the study from a parent or a legal guardian, and iii) not affected by a psychiatric disorder incompatible with the correct understanding of the research questions. | Thematic content analysis |
| Burton et al. 2024^59^, USA | In-person Focus groups | New Moms, a non-profit organization on the West Side of Chicago | All pregnant women attending prenatal classes at New Moms were approached between March 2019 and June 2019 | Thematic analysis |
| Cumming and Symon, 2025^46^, UK | Semi-structured interviews (mixture of in-person, telephone or online) | Snowball sampling to identify contacts with five UK Third Sector Organisations (TSOs) who support homeless families located in London, South England, Northwest England and two cities in Scotland.  TSOs gave eligible participants the Participant Information Sheet (PIS)and completed a ‘permission to refer’ form that allowed contact details and preferred language to be shared with the lead researcher. | Aged over 16, between 28 weeks pregnant and 24 months postnatal, and identified as homeless during pregnancy.  Pregnant mothers with known Child Protection Orders to re-move the baby at birth were not included for ethical reasons as some interview questions focused on parenting and feeding intentions. | Reflexive Thematic Analysis |
| Dundas et al. 2023^60^, UK | In-person semi-structured interviews | Study participants were recruited from 2 population-level health surveys featuring >10000 residents of Scotland and UK. Additional interview participants were recruited from community groups that support low-income families | Based on eligibility for the Healthy Start Voucher (HSV) scheme (i.e. income-based criteria devised by UK and Scottish government) as follows: (1) Growing Up in Scotland (GUS) mothers who received HSVs; (2) GUS mothers eligible for HSVs but who did not claim; (3) mothers who were receiving HSVs; (4) mothers who were eligible for HSVs but who did not claim; (5) mothers who were nearly eligible for HSV, but did not meet the criteria. | Thematic analysis |
| Ellul et al. 2020^61^, UK | In-depth interviews | Women purposively recruited via local voluntary sector groups, word-of-mouth, and through friends and peers. | Forced migrant women who had been destitute at some point in their pregnancy, were over 18 years old and had a baby within the last 2 years. Women who had an adverse pregnancy outcome were not included | Thematic analysis |
| Evenosky et al. 2021^62^, USA | Semi-structured interviews using a moderator guide followed by a short demographic / descriptive survey | Pregnancy support groups at a non-profit social service agency in Norristown | Norristown residents and be currently pregnant or have given birth within the previous 12 months, and English-speaking | Thematic analysis |
| Gewalt et al. 2019^63^, Germany | Semi-structured, repeat interviews | State provided direct provision accommodation/reception centers for pregnant asylum seekers | Pregnant asylum seekers in first or second trimester living in state provided direct provision accommodation who had come to Germany 1-6 months prior | Thematic analysis |
| Graham et al. 2016^47^, USA | Individual and group semi-structured and structured interviews | Healthy Start Program, Child Care Council or at the Centre for Community Health, Rochester (NY) | Low-income women, age 18-35, living in greater Rochester (NY) and eligible for the Special Supplemental Nutrition Program for WIC or PCAP | Thematic analysis and constant comparative method utilising hand coding and Excel spreadsheet to build integrative model |
| Gross et al. 2019^43^, USA | Semi-structured interviews | Large urban public hospital, New York City; embedded within an early child obesity prevention study | Pregnant Hispanic/Latina women over 18 yo in third trimester with a singleton pregnancy, fluent in English or Spanish and intended to receive care at study sites | Iterative process of textual analysis |
| Groth et al. 2016^48^, USA | Semi-structured in-depth interviews | University obstetric clinic in a medium-sized urban northeastern city | Aged 18 years or older, pregnant, self-identified as African American, and low-income as determined by medical insurance type or WIC eligibility. | Directed content analysis |
| Hromi-Fiedler et al. 2016^65^, USA | In-depth interviews collected as part of a larger study | Women's Ambulatory Health Services (WAHS) clinic at Hartford Hospital, Connecticut | Over 18 yo, in 2nd or 3rd trimester of pregnancy, WIC-eligible, Latina, having a singleton pregnancy, overweight or obese, not on a restricted diet and non-smoker | Thematic analysis |
| Iqbal et al. 2024^66,^ UK | Semi-structured interviews | Women part of the Born in Bradford Better Start (BIBBS) study, women recruited at a routine appointment while awaiting their glucose tolerance test, offered to all women in Bradford between 26–28 weeks gestation to diagnose gestational diabetes | Pregnant Pakistani women aged over 18 years living in Better Start Bradford areas (defined by full postcode) who are registered to give birth at Bradford Teaching Hospitals NHS Foundation Trust (BTHFT). English speaking and reading (due to time constraints) | Inductive thematic analysis |
| Marshall et al, 2026^49^, UK | Semi-structured interviews | Lambeth, South London; recruited through community gatekeepers and extensive outreach methods including distributing leaflets in community centres, foodbanks, markets accepting food subsidy vouchers, and targeted social media groups; snowball sampling also employed. | Being 18 years or older, residing in Lambeth with recourse to public funds, being pregnant or having a child under one year, speaking English, and experiencing food insecurity. | Reflexive thematic analysis |
| McKerracher et al. 2020^67^, Canada | Focus group discussions | Locally administered prenatal programmes or as part of the Mothers to Babies (M2B) study follow-up in neighbourhoods characterised by high rates of poverty within Hamilton, Canada | Not reported | Thematic network analyses |
| Nagourney et al. 2019^68^, USA | Semi-structured, in depth interviews containing open-ended questions | Referral clinic for obese pregnant women at a large urban hospital in Baltimore, USA | Women in first or second trimester of pregnancy, aged 18 or over, and who had not yet received nutritional counselling | Topical codes were developed using Grounded Theory and analysis used the Social Cognitive Theory framework |
| Ohly et al. 2018^69^, UK | Combination of interview techniques: first interviews were semi-structured and then continued with open, realist questions | Two areas of North West England; face-to-face recruitment through midwifery services (antenatal clinics) and drop-in sessions (such as breastfeeding support groups) in Sure Start children's centres located in deprived areas. | Pregnant and receiving Healthy Start Vouchers or if they had been pregnant within 6 months and received Healthy Start vouchers during that pregnancy | Thematic analysis underpinned by realist methods |
| Oresnik et al, 2025^50^, Canada | Focus groups and one-to-one interviews | Focus groups with pregnant and postpartum people living in the City of Hamilton, Ontario, conducted as part of the Mothers to Babies Study (M2B); augmented with interviews with pregnant or recently postpartum people living in Hamilton who experienced household food insecurity (HFI), Gestational diabetes mellitus (GDM) or Anxiety and mood disorders (AMD) to further understand the potential syndemic interactions between these conditions.  Recruitment for one-on-one interviews advertised through connections with local midwifery groups and other pregnancy/postpartum support groups, including via social media pages (Facebook and Instagram) as well as health practitioners informing patients about the study. | Not explicitly reported. | Thematic analysis |
| Paul et al. 2013^70^, USA | Focus groups | Rochester, NY, advertised through flyers at obstetrics practices, Child Care Council, Inc. of Rochester, and a local health and parenting program for low-income women. | Pregnant women of both high and low income. Income cut off was women who were eligible for WIC and/or Prenatal Care Assistance Program (PCAP) | Kruger's note-based analysis method. A constant comparative method was used to code focus group notes and to identify additional emergent themes. |
| Quintanilha et al. 2018^51^, Canada | Semi-structured interviews | Community based participatory approach through the Multicultural Health Brokers Cooperative, Edmonton | Immigrant Somali women, identified by health broker, who were pregnant or postpartum and food insecure | Qualitative content analysis |
| Reyes et al. 2013^71^, USA | Semi-structured interviews | Mothers in the waiting room of a single university-affiliated outpatient prenatal care clinic in Philadelphia which served primarily Medicaid insured patients | Mothers who self-identified as African American, were at least 18 years old, and received Medicaid (income proxy) | Principles of grounded theory. Two authors independently coded the data to identify themes and used Atlas.ti software to assist with data coding and management |
| Sim et al. 2020^52^, Canada | Semi-structured, repeat interviews conducted in person | Birthing centre of IWK Health Centre in Halifax, Nova Scotia, Canada | Resident of Halifax, over 18 yo, first (singleton) pregnancy, Pre-pregnancy BMI<27 kg/m2, stated intention to breastfeed, monthly income at or below LICO cut-off and responded true to either of the following statements based on the past 12 months 1) worry about whether food would run out before we got money to buy more or 2) food bought did not last and not have enough money to get more | Iterative, Foucauldian and feminist discourse analysis methods |
| Struthers et al. 2019^72,^ Canada | Case study approach using multiple sources – public records, program documents and interviews (in-person and via telephone). | Manitoba, Canada. Recruited with support from HBPB staff, via an invitation to participate in the  study in information letters. Participants either responded to the invitation or  heard about the research from a friend or family member who had received the invitation. | Recipients of the Healthy Baby Prenatal Benefit | General inductive approach using qualitative analysis software |
| Waberi et al, 2025^53^, Denmark | Focus groups and interviews, supplemented by ethnographic notes and reflections | Aarhus' ‘Parallel Societies / Transformation Neighbourhoods’, recruitment through two rounds of snowball sampling, through NGOs, branches of municipal organs for early childhood education and public health, and a midwifery clinic. | Not explicitly stated | Thematic network analysis |
| Wise et al. 2015^73^, USA | Focus groups | Prenatal clinic, a school-based adolescent parenting program, and a high school in the Mid-Atlantic region | Participants were fluent in English, and all participants were currently attending high school | Data coded and analysed thematically using methodology by Morrison-Beedy, Cote-Arsenault, and Fischbeck Feinstein for focus group analysis |
| Whisner et al. 2016^74^, USA | Administered Questionnaire | Rochester Adolescent Maternity Program | 18 yo or younger at time of enrolment, 12 or more weeks of gestation at time of enrolment and singleton pregnancy.  Exclusion criteria included current or previous diagnosis of HIV, diabetes, malabsorption diseases, and eating disorders. | Thematic analysis |
| Yee et al. 2016^54^, USA | Repeat, in-person semi-structured interviews | Outpatient prenatal clinic of a large urban academic medical centre; Participants were identified via clinical care and were invited to participate by in-person interaction with a member of the research team. | English-speaking, over 18 yo, pregnant with GDM or T2DM before 30 weeks gestation | Qualitative data analysis including coding, data management and text retrieval using ATLAS.ti 6 software. |
| Zinga et al. 2022^55^, Australia | Semi-structured telephone interviews using a 28-question schedule | Australia’s largest maternity hospital, the Royal Women’s Hospital in Melbourne | Pregnant attending antenatal clinics at RWH, English speaking, experiencing financial hardship and deemed to be food insecure (by receipt of government income benefit, use of food programs or affirmative answer to at least one food security assessment item) | Inductive, thematic analysis. |

**Table S5: Description of themes and sub-themes across included studies exploring pregnant women’s experiences of food insecurity in high-income countries (HICs) and CERQual assessment of confidence (n=32)**

| **Subtheme** | **Experiences** | **Further evidence** | **CERQual assessment of confidence in evidence** | **Explanation of CERQual assessment** |
| --- | --- | --- | --- | --- |
| **Theme one: Barriers in access to food** | | | | |
| **Economic constraints** | - Fresh fruit and vegetables and meat are too expensive - Women can’t afford a healthy diet - Prioritization of long shelf life, filling and cheaper cupboard foods - High food prices lead to feelings of a loss of cultural identity - Trade-offs with other material resources such as electricity and fuel - Constrained access to employment opportunities and income | - *“I try and make my food shopping as – like, the produce – as fresh as possible, but again, you’ve just gotta be cautious that that does cost more money than the pasta and sauces, and it’s just so much cheaper to go the other way”*(Zinga, 2022)^55^ - *“Healthy food’s not cheap. The ‘crap food’ is cheaper than healthy food, which is completely backwards. They want you to eat all this healthy stuff, but they make it impossible for [lower]-income families to get that. And then you go to the food bank and if you’re lucky they have some produce [that’s] kind of, like, old”* (Oresnik, 2025)^50^ - *“I can’t afford to eat right”*(Yee, 2015)^47^ - “*being able to get he actual good, healthy food. Its so expensive compared to everything else”*(Yee, 2015)^54^ - *‘Mothers relied on inexpensive staples that stored easily and would not spoil, such as beans, rice, and tortillas’ (*Gross, 2019)^48^ - *“cause eating healthy is not that easy and I think it is more expensive, vegetables are just more expensive than getting junk food”* (Graham, 2016)^47^ - *“meat is something I just haven’t been able to afford or have access to”* (Zinga, 2022)^55^ - *‘rice, bread and pasta as the main foods they could afford…often they could not buy fruit, vegetables and meat’* (Quintanilha, 2019)^51^ - *“I eat better when I ﬁrst get my food stamps. I can buy my fruits, I can buy my vegetables... I won't eat good again until next month because I can't buy fruits and vegetables fresh all the time”* (Hromi-Fiedler, 2016)^65^ - *“Even when I tried looking for doing something from home, I couldn’t get anything … I looked for a part-time job, but I couldn’t find anything that fit in with school”* (Marshall, 2026)^49^ | **High***** | No or very minor concerns in all four domains |
| **Physical and environmental barriers** | - Women relied on walking or public transport to food shop - Big supermarkets are cheaper but harder to access - Transportation was a barrier to healthy eating | - *‘majority of mothers did not have cars and used public transportation to get around,’* (Reyes, 2013)^71^ - ‘*the ability to buy food from town supermarkets and stock up the pantry several times a year was helpful’*(Booth, 2023)^45^ - *“Obviously, I can feed us takeout on a fiver, rather than spend a lot more on healthy food they have there that I can’t really afford.”* (Iqbal, 2024)^66^ |  |  |
| **Cyclic insecurity** | - Limited access to healthy food by the end of month - Run out of food end of the month - Constrained access to employment opportunities and income | - *“When it gets close to the end of the month it (healthy food) always seems to run out,”* (Reyes, 2013)^71^ - *“I need to make sure that I make that . . . last throughout the month.”* (Evenosky, 2021)^62^ - *“very end of the cycle before you get paid that you’ve got no bread, no milk, no – nothing in the pantry and you’ve got your last 30 dollars and you also need to try and get some petrol in there as well. It’s tough”* (Zinga, 2022)^55^ - *‘food stocks dropped to perilous levels at the end of their pay cycles’* (Zinga, 2022)^55^ - *“Toward the end of the month, it gets a little hairy, but for the most part, I’ve learned how to budget so that I don’t end up with no food.”* (Allen, 2023)^44^ |  |  |
| **Lack of control** | - Women do not have control over (their own and household) diet - Cost dictates food choice | - *‘as mothers, a low sense of control overlapped with women’s negative perceptions of the quality of their families’ diets’* (Quintanilha, 2019)^51^ - *“I don’t feel I have much control … I have been stressed for the last six months”* (Quintanilha, 2019)^51^. - “*I requested the manager to keep some food in my room because I get hungry at night…but they refuse…I was so upset… It is just food we are asking for nothing else”* (Arshad, 2018)^57^ - *“Mom likes me to eat what she cooks”* (Whisner, 2016)^74^ |  |  |
| **Theme two: Impact on mental and physical health** | | | | |
| **Diet is poor quality** | - Barriers in access to food mean women have to compromise on diet quality - Weight gain - Negative impact on body image | - “*I lived off pot noodles and tinned pineapple… but that ended up making me really sick*” (Cumming and Symon, 2025)^46^ - “*food in acceptable quantity but not quality”* (Quintanilha, 2019)^51^ - *‘unhealthier foods of minimal quality were frequently consumed’* (Sim, 2020)^52^ - *‘maintaining their weight within the normative standard was challenging under resource constraint’* (Sim, 2020)^52^ - ‘*excess maternal weight was a failure on both personal and moral level’* (Sim, 2020)^52^ | **High***** | No or very minor concerns in all four domains |
| **Hunger** | - Limiting portion sizes and missing meals - Physical pain - Exacerbated impacts for additionally marginalised populations such as migrant women, women in detention and in recovery | - ‘*frequently skipping meals*’ (Graham, 2016)^47^ - “*The only thing that would help me would be to snack every couple of hours . . . because if I’m vomiting from the very beginning of the day, I don’t keep my medicine down . . . but I can’t always afford or get my medicine for nausea. So, if I couldn’t do that, I’d have to rely on being able to snack every couple of hours. That would keep the nausea down . . .because I have to be able to keep down my medicine (methadone) from the clinic, otherwise I’d be in withdrawal*” (Allan, 2024)^43^ - *“I try to save with food, for example, we try not to eat excessively, like large quantities”* (Gross, 2019)^48^ - “*sometimes I would live for the whole day, I didn’t eat*” (Ellul, 2020)^61^ - *“guts are hurting”* (Booth, 2023)^45^ - *‘physical pain and emotional distress were recounted as a consequence of hunger’* (Booth, 2023)^45^ - *all the women felt hungry in between meals, especially in the evenings and were not allowed to take any food back to their room to snack’* (Arshad, 2018)^57^ |  |  |
| **Concern for newborn health** | - Pregnancy diet is important for newborn health - Worry newborn will not get the right nutrients - Breastfeeding worries | - *‘women believed that poor prenatal F&V intake caused suboptimal fetal growth and brain development … birth defects, poorer immune system and smaller infants at birth’* (Hromi-Fiedler, 2016)^65^ - “*What I have to eat—am I going to be able to get enough nutrition to be sufficient for him?”* (Sim, 2020)^52^ - ‘*food insecurity was influencing her breastmilk’* (Sim, 2020)^52^ - *‘because of fears that their own poor diet would adversely affect breast milk quantity and quality’* (Gross, 2019)^48^ - *‘I would only eat twice a day, sometimes only bread. It's very difficult to have been pregnant in a hotel. When you're hungry overnight and you're not able to eat, I would feel my baby was hungry too’* (Cumming and Symon, 2025)^46^ - *‘The beginning was so bad … I was losing weight instead of gaining weight. Now I’m little bit better. But my midwife said still the baby’s kind of small so you have to take like vitamin D, iron, calcium, prenatal vitamins’* (Oresnik, 2025)^50^ |  |  |
| **Stress** | - Constant worry about food - Pregnancy exacerbates stress - Other bills to pay | - *“I don’t feel I have much control … I have been stressed for the last six months”* (Quintanilha, 2019)^51^ - *“a constant concern… it’s always on my mind”* (Zinga, 2022)^55^ - *‘worried about money all the time’* (Ohly, 2018)^69^ - *“it’s already a big family, we are four here”* (Gross, 2019)^48^ |  |  |
| **Theme three: Established coping strategies** | | | | |
| **Individual strategies** | - Preplanning meals and budgeting - Coupons, food nearing its sell-by-date and creativity/substitution of ingredients - Children eat first - Culturally specific and diverse strategies to support access to food | - *various strategies to stretch the food available as much as possible in a month’* (Quintanilha, 2019)^51^ - *active limiting.. a tool to prevent the family from not having enough food the next day’* (Gross, 2019)^48^ - *‘meal planning and purchasing to occur within a strict budget’* (Zinga, 2022)^55^ - *“At the end of the market they usually have really cheap … they are not fresh … but they are a bit cheaper”* (Marshall, 2026)^49^ - *“my kids will eat and I’ll just be stuck”* (Yee, 2015)^54^ | **Moderate**** | Moderate concerns regarding adequacy; no/very minor concerns across remaining three domains. |
| **Informal support and reliance on others** | - Rely on family, friends and neighbours for food and cooking facilities - Help with transport | - *“we usually like have to go ask the in-laws and stuff like that for a top-up”* (Booth, 2023)^45^ - *“when we don’t have enough food I …go ask around family members”,* (Booth, 2023)^45^ - *‘were not responsible for shopping or cooking’* (Paul, 2012)^70^ |  |  |
| **Food assistance programmes and support from healthcare professionals** | - Food assistance programs as welcome and crucial - Vouchers help afford fruit and veg - Vouchers do not last through month - Barriers in access to food assistance - Social stigma | - *“It just helps me by like healthy food is expensive, more expensive than junk food amazingly enough, so it just helps with getting fresh fruit and vegetables and stuff into my fridge”* (Struthers, 2019)^72^ - *“run out by the end of the month”* (Gross, 2019)^48^ - *“not the type of person to throw [her] entire life out there” and worried about being “judged by that opposing person.”* (Allen, 2023)^44^ - *“got them and then they just like stopped suddenly, just kinda like with, round about like when she was due... then I mentioned it to the health visitors like about what to do and she said that sometimes they just kinda like stop and then I have to kinda apply for it again"* (Dundas, 2023)^60^ - *‘we frequently observed dozens of mothers jostling for and worrying about access to weekly food handouts at a local NGO; on three occasions, the senior author observed young children hanging around a local grocery store bakery complaining of hunger and asking for free ‘child buns’* (Waberi, 2025)^53^ |  |  |

***** It is highly likely that the review finding is a reasonable representation of the phenomenon of interest**

**** It is likely that the review finding is a reasonable representation of the phenomenon of interest.**
